# Spray-Assisted Interfacial Polymerization to Form Cu^II/I^@CMC-PANI Film: An Efficient Dip Catalyst for A^3^ Reaction

**DOI:** 10.3390/nano12101641

**Published:** 2022-05-11

**Authors:** Zhian Xu, Liang Xiao, Xuetao Fan, Dongtao Lin, Liting Ma, Guochao Nie, Yiqun Li

**Affiliations:** 1 Department of Chemistry, College of Chemistry and Materials Science, Jinan University, Guangzhou 511443, China; zhian_xu@outlook.com (Z.X.); liang_xi728@163.com (L.X.); lovefandou1018@gmail.com (X.F.); Dongtao_Lin@163.com (D.L.); 2 Photoelectric Information Center, School of Physics and Telecom, Yulin Normal University, Yulin 537000, China; mikt13f@163.com

**Keywords:** interfacial polymerization, spraying method, carboxymethylcellulose, polyaniline, copper catalyst, dip catalyst, A^3^ reaction

## Abstract

A novel and interesting method for the preparation of carboxymethylcellulose–polyaniline film-supported copper catalyst (Cu^II/I^@CMC-PANI) has been developed via spray-assisted interfacial polymerization. Using copper sulfate as an initiator, spraying technology was introduced to form a unique interface that is perfectly beneficial to the polymerization of aniline monomers onto carboxymethylcellulose macromolecule chains. To further confirm the composition and structure of the as-prepared hybrid film, it was systematically characterized by inductively coupled plasma (ICP), Fourier transform infrared spectroscopy (FTIR), X-ray photoelectron spectroscopy (XPS), X-ray diffraction (XRD), scanning electron microscopy (SEM), energy-dispersive X-ray spectroscopy (EDS), and thermogravimetric analysis (TGA) techniques. The Cu content in the fresh Cu^II/I^@CMC-PANI film was determined to be 1.805 mmol/g, and spherical nanoparticles with an average size of ca. 10.04 nm could be observed in the hybrid film. The Cu^II/I^@CMC-PANI hybrid film was exerted as a dip catalyst to catalyze the aldehyde–alkyne–amine (A^3^) coupling reactions. High yields of the products (up to 97%) were obtained in this catalytic system, and the catalyst could be easily picked up from the reaction mixture by tweezers and reused for at least six consecutive runs, without any discernible losses in its activity in the model reaction. The dip catalyst of Cu^II/I^@CMC-PANI, with easy fabrication, convenient deployment, superior catalytic activity, and great reusability, is expected to be very useful in organic synthesis.

## 1. Introduction

In 2010, a pioneering researcher Radhakrishnan developed the concept of “dip catalyst”, and successively presented an Ag nanoparticle-embedded PVA thin film for the reduction of 4-nitrophenol by sodium borohydride [1]. Since then, the dip catalyst, which can switch the reaction on and off instantaneously, by merely dipping in and out of the reaction vessels, has drawn increasing attention for its ease of fabrication, excellent catalytic performance, convenient separation and reusability, and environmental friendliness [2]. To date, the dip catalyst has emerged as one of the most powerful tools in catalysis, synthetic methodology, materials science, and environmental science. A considerable number of dip catalysts have been well documented in the last dozen years, and the range of reported dip catalysts includes Pt@GS (dip coating) [3], CMC-Ni-BC (coating) [4], gold nanoparticle-loaded filter paper (impregnated into the filter paper) [5], palladium nanoparticle-loaded cellulose paper (dip coating) [6], Pd@filter paper (dip coating) [7], Pt-PVA thin film (spin coating) [8], Pd-PVA (spin coating) [9], AgNPs@NH_2_-CP (chemical modification) [10], Cu@CS-FP (layer coating) [11], Ag/CH-FP (layer coating) [12], and so on. Common strategies for the fabrication of a dip catalyst previously included dip-coating [3,4,5,6,7] and spin-coating technology [8,9], chemical modification of thin films with a variety of functional units [10], and layer coating over thin films with functional material [11,12]. However, these preparation procedures suffered from one or more drawbacks, such as tedious operation, being time consuming, involving multiple step reactions, and possessing low catalytic performance. Therefore, searching for novel and convenient protocols for the fabrication of a dip catalyst with excellent catalytic activity and reusability is still in great demand.

Copper is a low-toxicity, economical, sustainable, and readily available metal elemental, belonging to the [Ar]^3^d^9^ transition metal, with various oxidation states, such as Cu(0), Cu(I), Cu(II), and Cu(III). Depending on its unique properties and characteristics, copper can effectively catalyze various organic reactions, such as Suzuki–Miyaura cross-coupling reactions [13,14], azide–alkyne cycloadditions [15], and Ullmann-type coupling reactions [16,17]. Performing the transition metal-catalyzed three-component reactions of aldehydes, alkynes, and amines (commonly called A^3^ reactions) in an atom-economic way is of importance, as it produces valuable N-containing heteroatom propargylamine products [18]. A great number of transition metal catalysts, including Cu, Ag, Au, and so on, have been exploited for the A^3^ coupling process. Several reviews have outlined the latest progress in A^3^ couplings well, and a large variety of copper catalysts have been criticized in these surveys [18,19,20]. However, there are no pertinent reports of a copper-based dip catalyst being applied to a one-pot A^3^ coupling reaction for the synthesis of versatile propargylamines.

We envisaged that polymer thin films generated in situ by interfacial polymerization, initiated by metal salts and embedded metal catalysts simultaneously, would be a class of easily fabricated, efficient, and reusable dip catalysts. Interfacial polymerization is a process that utilizes the interfacial layer (liquid–liquid layer or liquid–gas layer, etc.) to provide a unique space to constrain the polymerization for the preparation of polymer films or membranes [21]. Polymer materials generated in situ by interfacial polymerization have multiple advantages, including mild production conditions, high molecular weight, excellent uniformity [21], and high permeation rates [22]. The interfacial polymerization method also permits the creation of films that are highly resistant to destruction by exposure to harsh environments [22]. These merits of the films prepared by interfacial polymerization make them beneficial to be a dip catalyst.

Polyaniline exhibits good affinity to metal ions, through the unique coordination with its nitrogen atoms [23] and delocalized π-π conjugate system [24]; thus, it is a useful metal supportive material to form PANI-based metal catalysts (Metal@PANI), such as Pd@PANI [25,26], Fe@PANI [27], and Ag@PANI [28]. However, as most developed catalysts were reported in the preparation of powder-based PANI hybrids, using PANI to form a film is difficult, due to its strong rigidity, even when using an advanced method, such as the interfacial polymerization method mentioned above. The traditional method for the preparation of PANI films is the solution casting technique, using various solvents, such as *m*-cresol solution [29], to dissolve PANIs. To develop a new approach, a template method was developed by using synthetic polymer film as a soft template for the polymerization of aniline monomer on its surface, and, thus, assisted the formation of PANI film [30]. Carboxymethylcellulose (CMC) is non-toxic, biodegrade, economic, and eco-friendly, and it bears a great number of carboxymethyl (–CH_2_COO^−^) and free hydroxyl (–OH) groups on its glucose-unit chain, providing it with a splendid capacity to coordinate with various metal cations [31], and to form H-bonds with molecules containing active groups, such as hydroxy-, amino-, and carboxylic groups.

Based on the properties of PANI and CMC, in our previous work, we fabricated CMC-PANI hybrids via the one-pot and one-step oxidative polymerization of aniline, with CMC as the soft template and CuSO_4_ as the initiator [32]. However, the CuSO_4_NPs@CMC/PANI hybrids were obtained as dark green powders, which are not appropriate for application in dip catalysis. Interfacial polymerization may be an alternative approach to address the PANI shaping issue. Unfortunately, many attempts made on interfacial polymerization by various conventional methods, including pouring, dropping, immersing, and so on, failed to obtain the desired CMC-PANI hybrid film. It was reported that the occurrence of interfacial polymerization needs an interface between two phases to provide a unique reaction space [33,34]. However, the interfacial polymerization of aniline monomer onto the surface of the CMC-Na macromolecular chain is hard to perform using CuSO_4_ solution as an initiator, by conventional approaches, such as pouring, dropping, or immersing methods, as these two phases are both water soluble. Creatively, the spraying method was introduced into the process to solve this problem. By spraying copper sulfate solution onto the surface of the CMC and aniline mixture, aniline monomers polymerized immediately to rapidly form an extremely thin layer of CMC-PANI film. This thin layer served as an interface to provide a suitable space for further interfacial polymerization. Concerning the above facts, innovatively, we have attempted to fabricate carboxymethylcellulose–polyaniline film-supported copper catalyst (Cu^II/I^@CMC-PANI) in situ via spray-assisted interfacial polymerization, and further explored it as a dip catalyst for A^3^ reactions.

## 2. Experimental

### 2.1. Materials and Instrumentations

All chemicals were purchased from commercial sources and used as received, without further purification.

Gas chromatography (GC) was performed on a Shimadzu GCMS-QP2020 (Kyoto, Japan). The copper content of the catalyst was determined by inductively coupled plasma atomic emission spectrometry (ICP-AES), using a Thermo Fisher Scientific X Series 2 instrument (Waltham, MA, USA). Fourier transform infrared spectra (FT-IR) were collected on a PerkinElmer FT-IR Spectrometer Spectrum Two (Waltham, MA, America) within the spectral range of 4000–400 cm^−1^. X-ray photoelectron spectroscopy (XPS) data were obtained on a Thermo Fisher Scientific K-Alpha instrument (Waltham, MA, America). X-ray powder diffraction (XRD) data were collected on a Rigaku MiniFlex600 diffractometer (Tokyo, Japan), using Cu Kα radiation in a range of Bragg’s angles (5°–80°). Scanning electron microscopy (SEM) and energy-dispersive spectroscopy (EDS) were conducted with a Zeiss Sigma 300 instrument (Oberkochen, Germany). Thermogravimetric analysis (TGA) and derivative thermogravimetric analysis (DTG) were performed on a Mettler TGA/DSC3+ (Zurich, Switzerland), under a nitrogen atmosphere from 30 to 800 °C in a 50 mL·min^−1^ N^2^ flow and at a ramp rate of 10 °C·min^−1^. ^1^H NMR (300 MHz) and ^13^C NMR (75 MHz) spectra were obtained with a Bruker 300 Avance instrument (Karlsruhe, Germany), with CDCl_3_ as the solvent and TMS as the internal standard. HRMS was determined by using Agilent 6545 Q-TOF MS (Santa Clara, CA, USA).

### 2.2. Preparation of Cu^II/I^@CMC-PANI Film

**Scheme 1:**
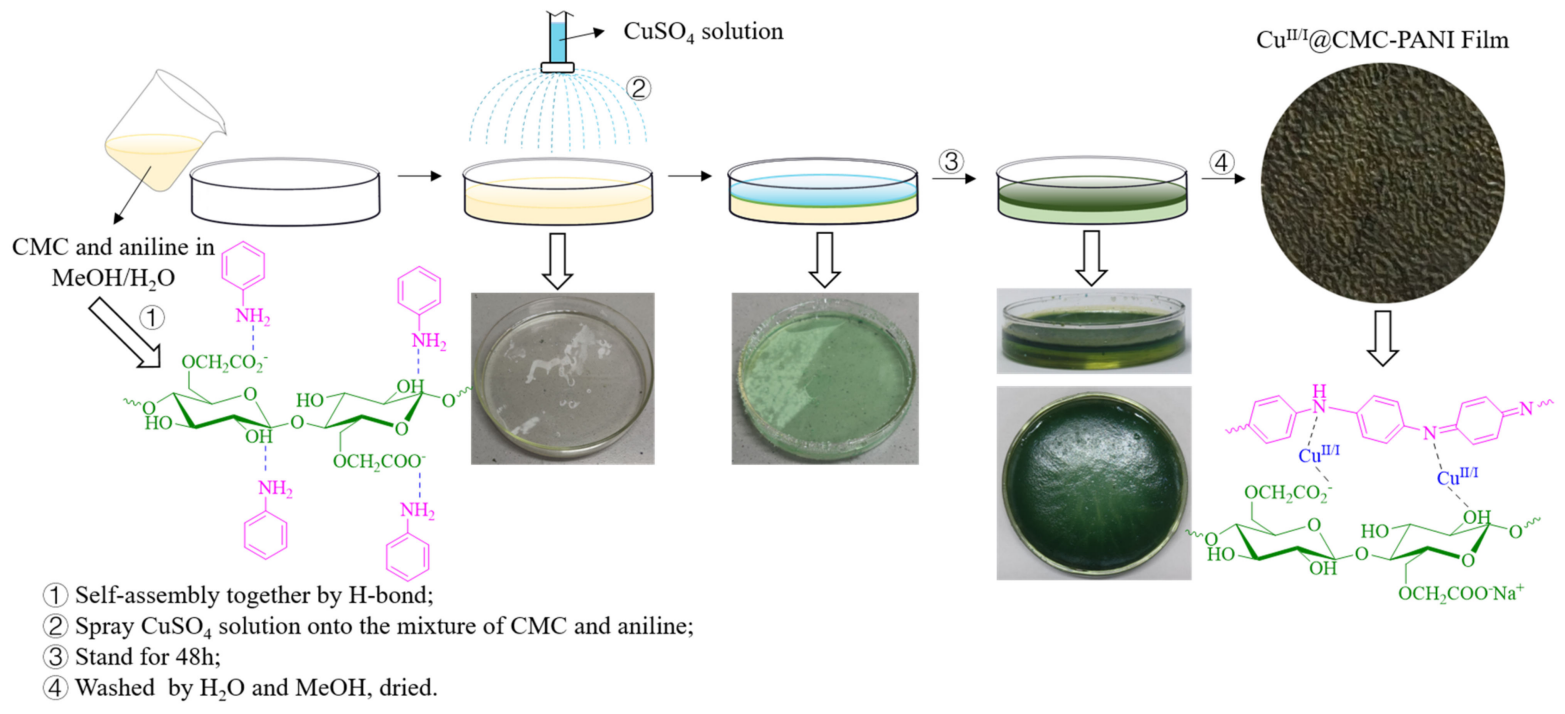
Schematic illustration of the preparation of Cu^II/I^@CMC-PANI film.

CMC-Na (0.242 g, 1 mmol) was added to 30 mL aqueous methanol solution (MeOH/H_2_O, *v*/*v* = 1/2) at room temperature, with continuous stirring until it completely dissolved. Then, aniline monomer (0.0911 g, 1 mmol) was dropped in above the solution to form a homogeneous mixture. This mixture was poured into a Petri dish, and CuSO_4_ solution (5 wt.%) was finely and evenly misted onto the surface of the mixture using a handheld sprayer. Immediately, a light green interface (extremely thin film) was formed and isolated the CMC-Na/aniline mixture from the CuSO_4_ solution. The resultant system was stood for 48 h to form a dark green film. The as-formed film was washed thoroughly with methanol and water to remove unreacted aniline and CMC-Na, and dried to afford the Cu^II/I^@CMC-PANI film. The schematic illustration of the preparation process of the Cu^II/I^@CMC-PANI film is shown in Scheme 1.

### 2.3. General Procedure for A^3^ Coupling Reactions Catalysed by Cu^II/I^@CMC-PANI Dip Catalyst

Aldehyde (1.0 mmol), amine (1.2 mmol), terminal alkyne (1.5 mmol) and a catalytic amount of Cu^II/I^@CMC-PANI film (15 mg, 2.7 mol% of Cu) were added to 2 mL toluene in a sealed vessel, and the mixture was rigorously stirred at 110 °C for the specific time and monitored by TLC. As the reaction was completed, the dip catalyst was picked up with tweezers and washed several times with ethyl acetate and dried at 80 °C for the next run. The remaining mixture was extracted with ethyl acetate (3 × 4 mL). Then, the combined organic phase was washed with brine and dried over anhydrous Na_2_SO_4_. After removal of the organic solvent by a vacuum rotary evaporator, the crude product was purified by column chromatography on silica gel to afford the corresponding propargylamine. All the products, except product (i), are known, and their ^1^H NMR and ^13^C NMR data were found to be identical to those reported in previous literature. The new compound (i) was fully characterized by FT-IR, ^1^H NMR, ^13^C NMR, and HRMS. All these data and spectra have been concluded in Supplementary Materials.

## 3. Results and Discussion

### 3.1. Synthesis and Characterization of Catalyst

**Scheme 2:**
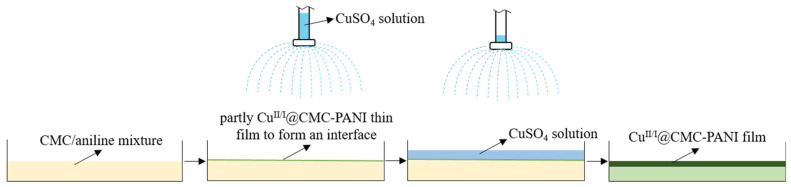
Detailed illustration of spray-assisted interfacial polymerization process.

The synthetic route of the Cu^II/I^@CMC-PANI film is outlined in Scheme 1. Before the addition of CuSO_4_ solution, the CMC-Na and aniline monomers were self-assembled together by H-bond interactions [35]. Then, the CuSO_4_ solution was finely and evenly misted onto the surface of the CMC-Na/aniline mixture, and a light green interface (an extremely thin film) was generated immediately, which separated the CMC-Na/aniline mixture and CuSO_4_ solution to provide an interfacial layer for aniline to polymerize (Scheme 2). In this process, the CuSO_4_ solution not only acted as the initiator, triggering the polymerization reaction, but also as the coupling reagent, combining the CMC molecular chain and PANI chain together. In the meantime, Cu(II)/Cu(I) species were deposited into the as-formed CMC-PANI film and stabilized by complexation with carboxylic groups (–COO^−^), hydroxyl groups (–OH), and nitrogen atoms (–NH– and –N=) to form the target dip catalyst. The Cu content in fresh Cu^II/I^@CMC-PANI film was determined by ICP-AES to be 1.805 mmol/g.

To verify the composition and structures of the Cu^II/I^@CMC-PANI film, analytical techniques, including FT-IR, XPS, XRD, SEM, EDS, TGA, and DGT, were performed.

**Figure 1:**
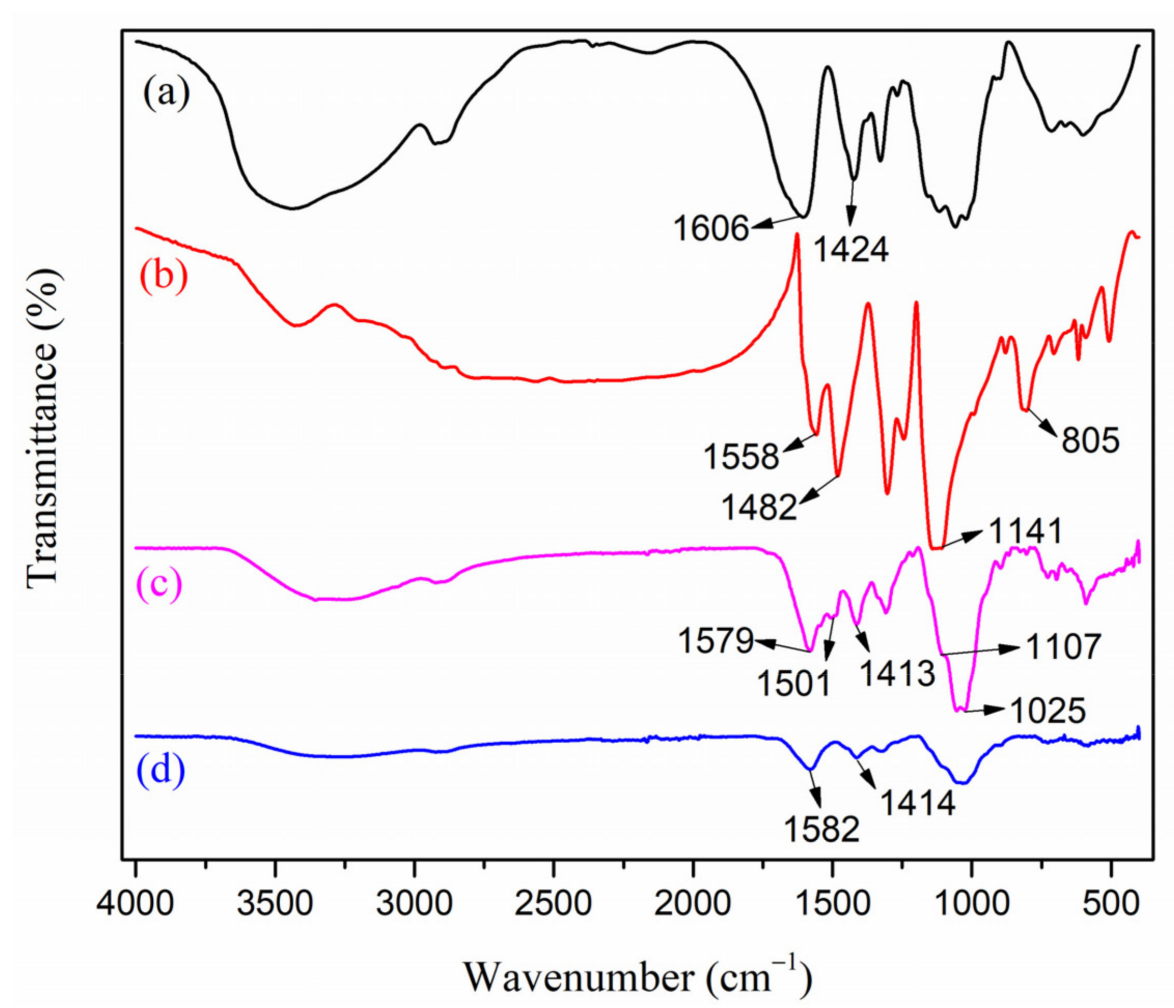
The FT-IR spectra of CMC-Na (a), PANI (b), the obverse side of Cu^II/I^@CMC-PANI film (c), and the back side of Cu^II/I^@CMC-PANI film (d).

The FT-IR spectra of CMC-Na (curve a), PANI (curve b), and the obverse side (curve c) and back side (curve d) of the Cu^II/I^@CMC-PANI film are presented in Figure 1. As shown in the spectrum of CMC-Na (Figure 1, curve a), the characteristic peaks appeared at 1606 cm^−1^ and 1424 cm^−1^, related to the asymmetric and symmetric stretching vibration of carboxylic (–COO^−^) groups, respectively [36,37]. In the spectra of PANI (Figure 1, curve b), the peaks at 1558 cm^−1^, coupled with 1141 cm^−1^, and 1482 cm^−1^, along with 805 cm^−1^, are assigned to the stretching vibration of quinoid and benzenoid rings, correspondingly [32,38]. Due to the interfacial polymerization, the Cu^II/I^@CMC-PANI film owns obverse and back sides, and they are composed of different components. The characteristic peaks of CMC (carboxylic groups: 1579 cm^−1^ and 1413 cm^−1^) and PANI (quinoid: 1107 cm^−1^ and benzenoid: 1501 cm^−1^) were both observed in the spectra of the catalyst obverse side (Figure 1, curve c), which confirmed that aniline monomers succeeded to the polymerization of PANI onto the CMC molecular chain. However, on the back side, only peaks of carboxylate groups (1582 cm^−1^ and 1414 cm^−1^) appeared (Figure 1, curve d), which means that PANI mostly exists on the obverse side of the catalyst.

**Figure 2:**
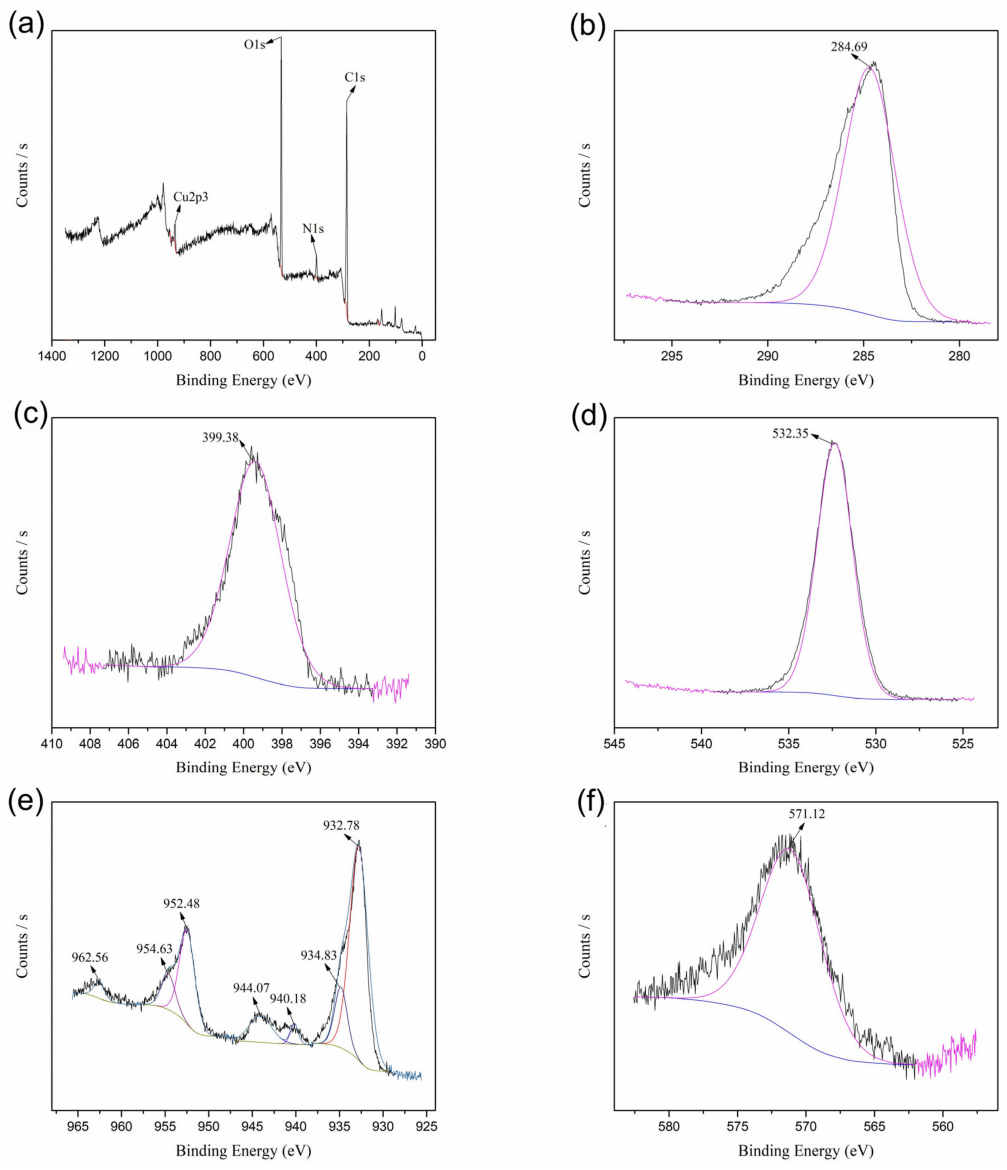

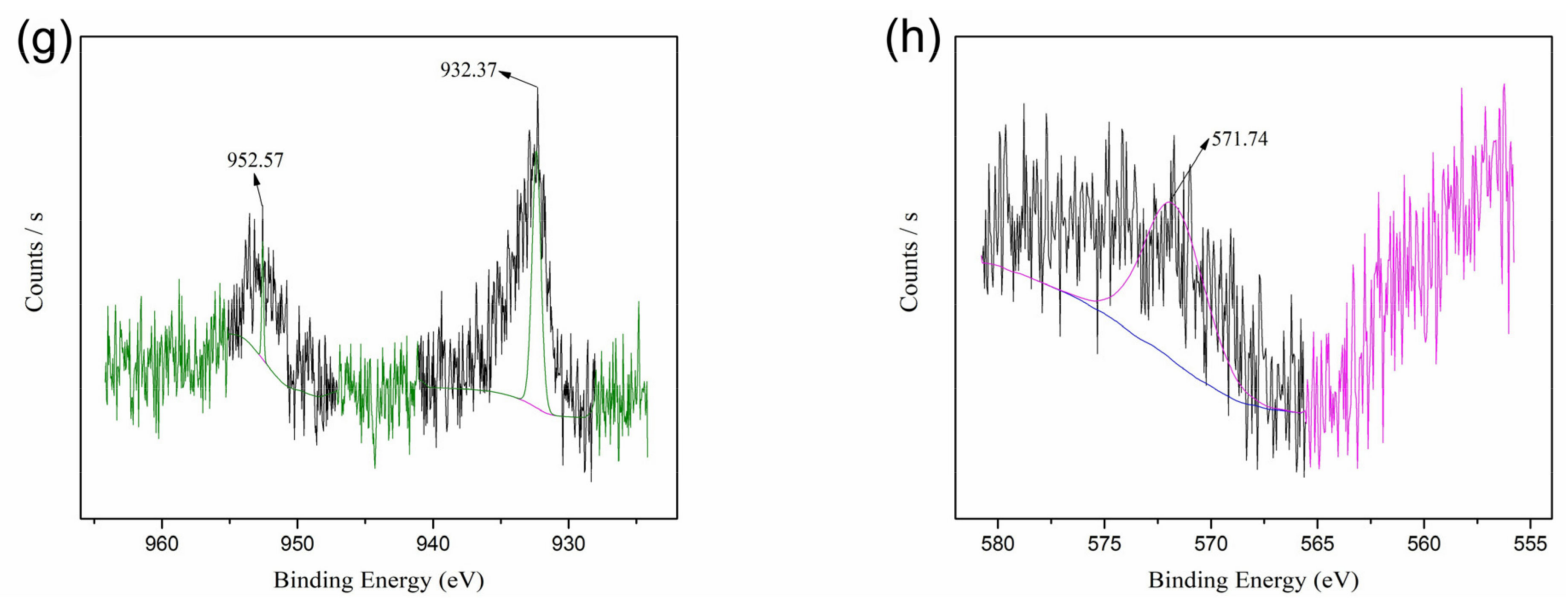
The XPS survey scan (**a**) of Cu^II/I^@CMC-PANI film and its high-resolution spectra of C_1s_ (**b**), N_1s_ (**c**), O_1s_ (**d**), Cu_2p_ (**e**) and Cu_LM2_ (**f**); XPS high-resolution spectra of Cu_2p_ (**g**) and Cu_LM2_ (**h**) of catalyst recovered from A^3^ reaction.

XPS was carried out to further demonstrate the existence of all elements and the oxidation state of copper. Presented in the survey scan spectrum (Figure 2a), C, N, O, and Cu elements are found to be 39.33%, 4.57%, 48.20%, and 7.90%, respectively, in fresh Cu^II/I^@CMC-PANI film. The peaks at 932.78 eV (Cu_2p3/2_) and 952.48 eV (Cu_2p1/2_) in Figure 2e, and the peak at 571.12 eV (Cu_LM2_) in Figure 2f, were assigned to the presence of Cu(I) [39]. Meanwhile, the shoulder peaks (934.83 eV and 954.63 eV) and satellites peaks (940.18 eV, 944.07 eV, and 962.56 eV) illustrate that Cu (II) also existed in the catalyst [40]. Notably, nearly all the Cu (II) in the recovered catalyst was turned into Cu(I), evidenced by the disappearance of shoulder peaks and satellites, as well as the presence of peaks at 932.37 eV (Cu_2p3/2_) and 952.57 eV (Cu_2p1/2_) in Figure 2g, and the peak at 571.74 eV (Cu_LM2_) [40] in Figure 2h, which means that Cu(I) species may be the true catalyst for A^3^ reactions.

**Figure 3:**
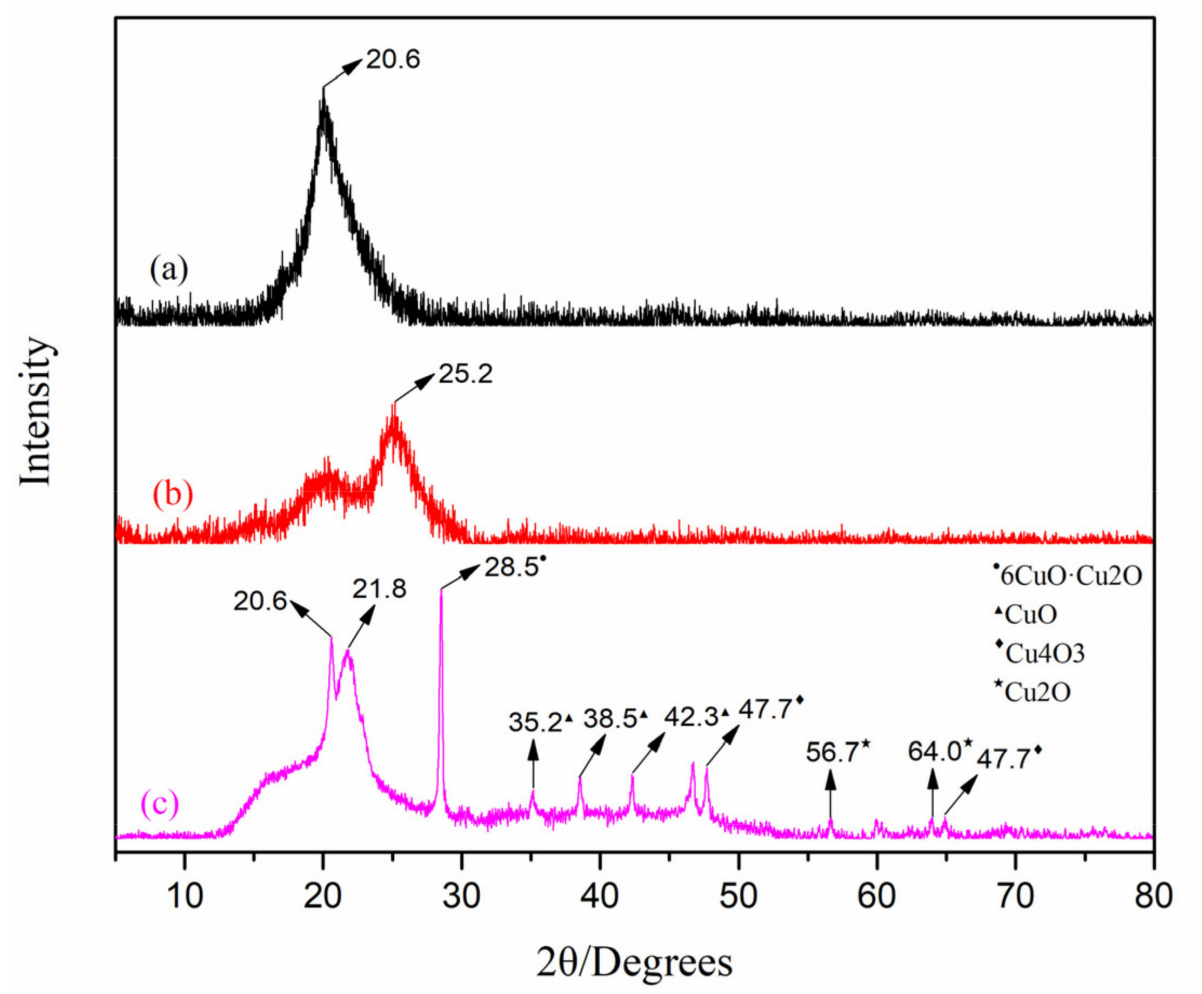
The XRD patterns of the CMC-Na (a), PANI (b), and Cu^II/I^@CMC-PANI film (c).

**Figure 4:**
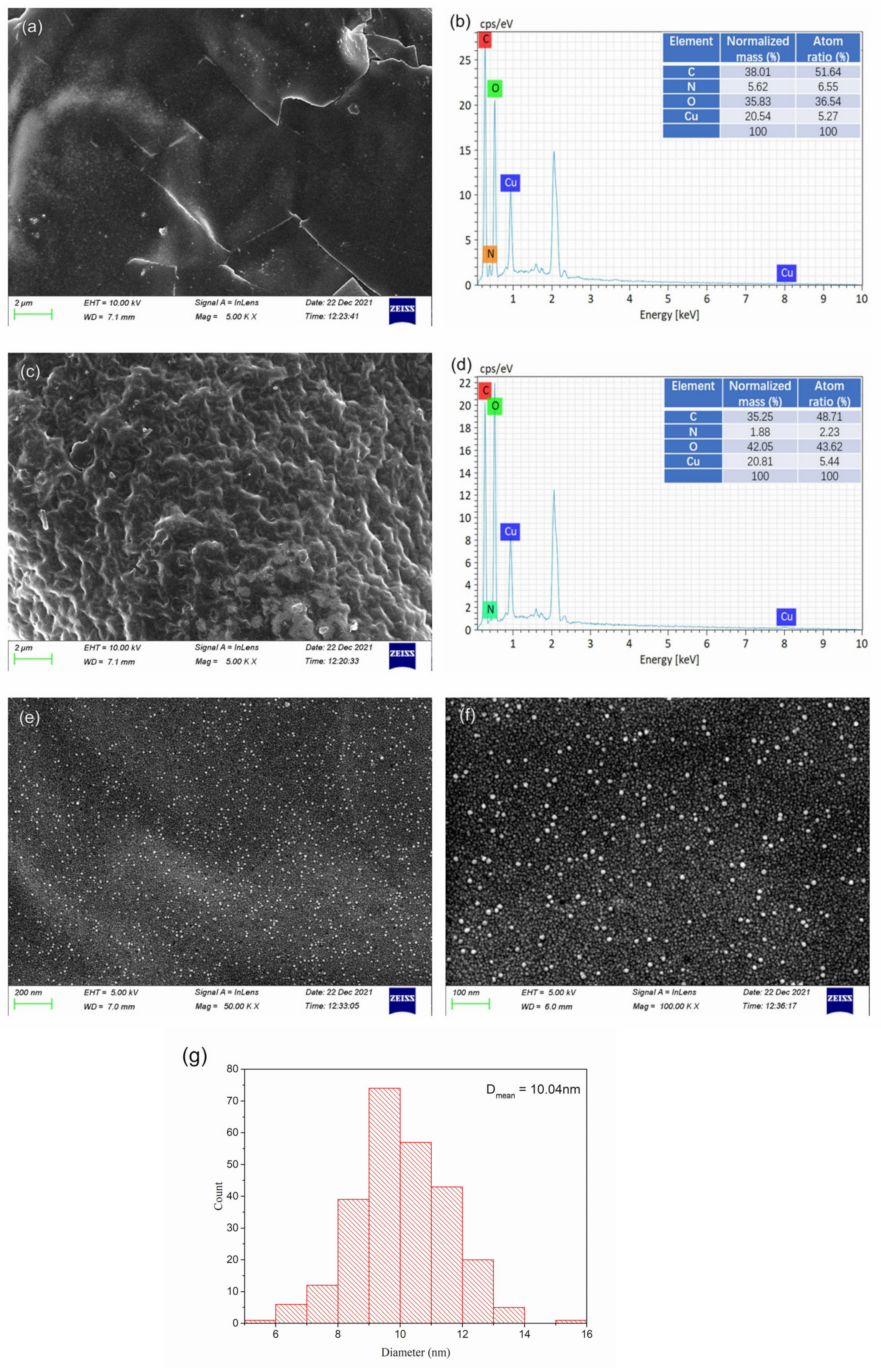
SEM images (**a**,**e**,**f**) and EDS data (**b**) of the obverse side of Cu^II/I^@CMC-PANI film; SEM images (**c**) and EDS data (**d**) of its back side; and histogram of particle size (**g**).

To further confirm the composition of the Cu^II/I^@CMC-PANI film, XRD was performed. The amorphous peak at 2*θ* = 20.6° in curve (a) and the broad peak at 2*θ* = 25.2° in curve (b) (Figure 3) were attributed to the pristine CMC-Na [41] and PANI powders [42], respectively, showing an extremely low degree of crystallinity. The peak at 2*θ* = 20.6° can also be observed in curve (c) of the catalyst, but the peak of PANI was shifted to 2*θ* = 21.8°, due to the interaction between PANI and CMC-Na [43]. The appearance of CMC and PANI peaks in curve (c) (Figure 3) proves that CMC and PANI were merged successfully into the film. Moreover, as can be found in curve (c) of the catalyst, the films consist of mixed phases of 6CuO·Cu_2_O (JCPDS 03-0879), CuO (JCPDS 44-0706), Cu_2_O (JCPDS 35-1091), and Cu_4_O_3_ (JCPDS 49-1830). Generally, the 6CuO·Cu_2_O phase is observed to be an intermediate phase between Cu_2_O and CuO [44], and Cu_4_O_3_ can be written as Cu(I)_2_Cu(II)_2_O_3_ [45]. These observations conclude that Cu (II) and Cu(I) species exist in the catalyst. The size of the particles can be calculated using the Scherrer equation, as follows: D = Kλ/(βcos*θ*) = $\frac{0.89\times 0.15405}{\frac{0.534}{180}\times 3.14\times \cos\frac{28.5}{2}}$ = 15.19 (nm). These data are approximate, with the value of 10.04 nm obtained from the SEM images (Figure 4e,f), due to the Scherrer equation being applicable to nanocrystals with perfect crystallinity, and there may be a certain number of errors to the particles without high crystallinity.

**Figure 5:**
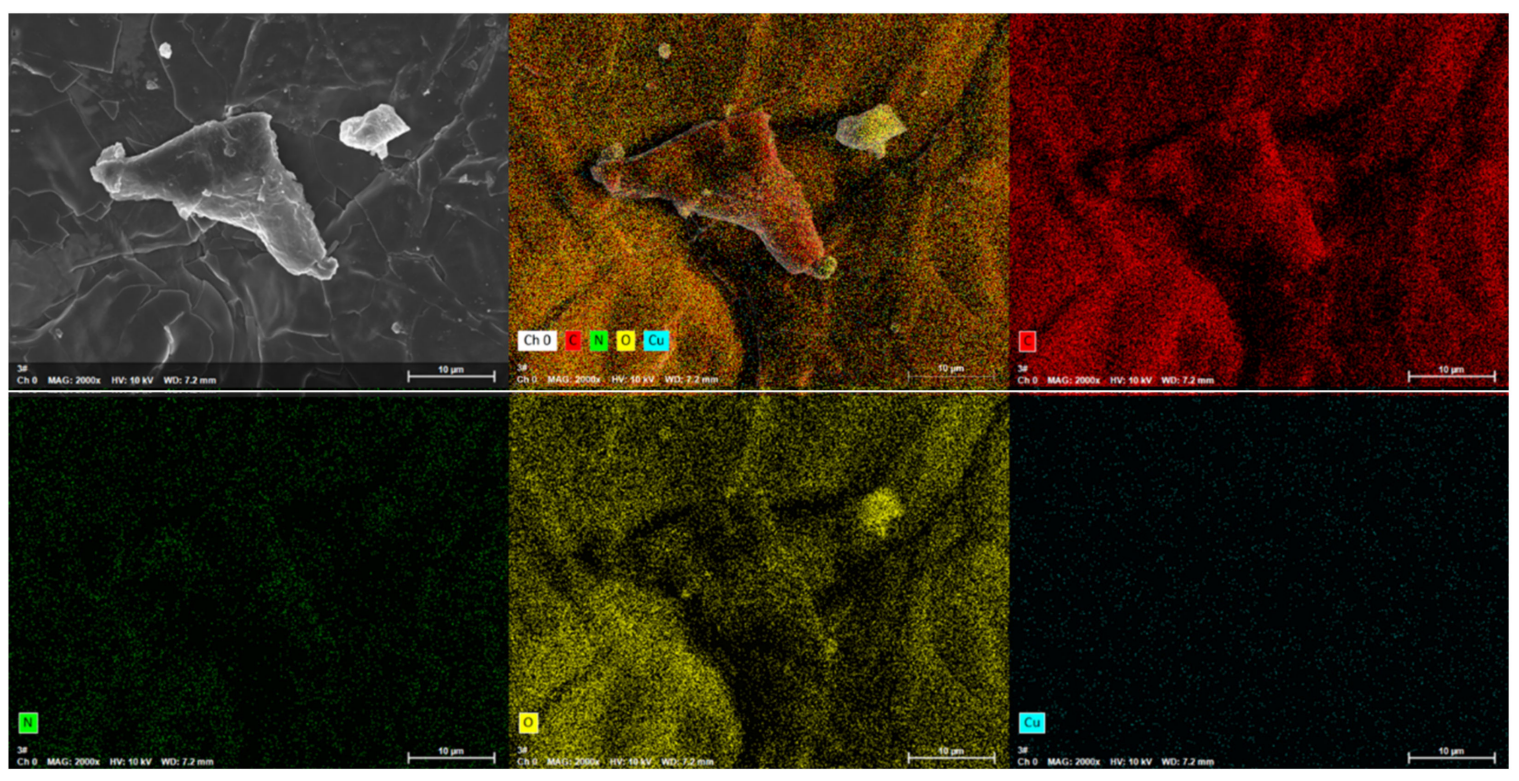
SEM corresponding elemental mapping images of Cu^II/I^@CMC-PANI film.

SEM and EDS were carried out in order to study the morphology and components of the Cu^II/I^@CMC-PANI film. The SEM images show a smooth surface for the obverse side (Figure 4a) and a rough surface for the back side (Figure 4c). It is worth noting that by increasing the magnification, spherical nanoparticles can be observed (Figure 4e,f), which may be attributed to the uniformly distributed copper oxides loaded in the film. The particle size histogram (Figure 4g) shows that the average particle size is approximately 10.04 nm in diameter. The EDS images of the obverse and back sides of Cu^II/I^@CMC-PANI are shown in Figure 4b,d. EDS clearly showed the presence of the nonmetallic elements C, N, and O, and the metallic element Cu in the dip catalyst. Significantly, the N content of the obverse side of the film (Figure 4b) is much higher than that of the back side (Figure 4d). This observation matched very well with the finding obtained from FT-IR (Figure 1), which illustrated that PANI mostly exists on the obverse side of the dip catalyst. The elemental mapping images (Figure 5), coupled with SEM, evidenced that the C, N, O, and Cu elements distributed throughout the catalyst in a homogeneous manner. The uniform distribution of Cu makes the catalyst work steadily.

**Figure 6:**
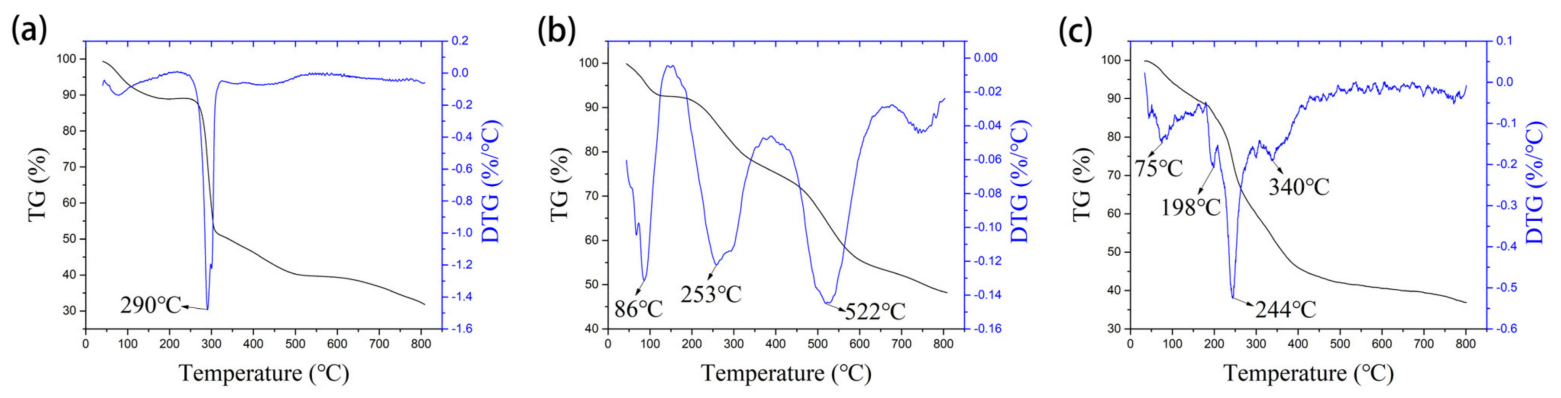
The TGA and DTG curves of CMC (**a**), PANI (**b**) and Cu^II/I^@CMC-PANI film (**c**).

To investigate the thermal behavior of the Cu^II/I^@CMC-PANI film, TGA and a corresponding DTG analysis of CMC-Na, PANI, and the Cu^II/I^@CMC-PANI film were performed, and the results are displayed in Figure 6. Below 100 °C, all the samples (Figure 6a–c) showed a slight mass loss, which can be attributed to the release of adsorbed moisture and volatile impurities. After that, CMC-Na (Figure 6a) presented a sharp mass loss at around 290 °C, which corresponds to the decomposition of its glucose-unit chain and side carboxylic groups [46]. PANI (Figure 6b) exhibited two sharp decreases in mass at around 253 °C and 522 °C, which may be caused by the deprotonation of PANI and decomposition of the backbone units of PANI, respectively [30,47]. In the curve of the dip catalyst (Figure 6c), a mass loss in the temperature range of 198–400 °C appeared. This may account for the combined influence of the decomposition of CMC and PANI chains. This result indicated that the dip catalyst is stable up to nearly 200 °C, and that it is applicable for further A^3^ reactions.

### 3.2. Application of Cu^II/I^@CMC-PANI Dip Catalyst in Three-Component A^3^ Coupling Reactions

First, the optimal reaction conditions for A^3^ reactions were explored in the presence of the Cu^II/I^@CMC-PANI dip catalyst (Table 1). Morpholine (1.2 mmol), *p*-chlorobenzaldehyde (1.0 mmol), and phenylacetylene (1.5 mmol) were selected as the model substrates. Initially, different solvents, including H_2_O, DMSO, DMF, CH_3_CN, EtOH, *n*-BuOH, and toluene, were screened to assess efficiency. It was found that the product yields increased with the decrease in solvent polarity. The highest yield was obtained when toluene was used as the solvent (Table 1, entry 7). Subsequently, to establish the fact that copper in the dip catalyst plays a key role in the A^3^ reaction, the model reaction was performed in toluene at 110 °C in the absence of a catalyst, and in the presence of 1.8 mol% and 2.7 mol% of Cu in the catalyst, the product yields obtained were 0%, 93%, and 97% (Table 1, entries 7–9), respectively. When further increasing the dose of the catalyst to 3.6 mol%, no significant elevation in the product yield was observed (Table 1, entry 10). These results suggested that copper is essential for this A^3^ model reaction, and 2.7 mol% of Cu is the optimal amount for the model reaction. Finally, the reaction temperature was explored, and it was found that decreasing the temperature from 110 °C to 90 °C, and even to 70 °C, had a considerable negative impact on the product yields (Table 1, entries 11–12). Therefore, it was concluded that the optimal condition involved morpholine (1.2 mmol), *p*-chlorobenzaldehyde (1.0 mmol), phenylacetylene (1.5 mmol), and the Cu^II/I^@CMC-PANI dip catalyst (2.7 mol% of Cu) in toluene at 110 °C.

Next, with the optimal reaction condition in hand, the substrate scope of the reaction was examined (Table 2). Aryl aldehydes, bearing both electron-withdrawing and electron-donating groups, proceeded well to afford excellent yields (Table 2, entries 1, 3, 4, and 6), while salicylaldehyde was difficult to conduct the reaction with (Table 2, entry 5), due to the intramolecular H-bond, which improves the stability of its aldehyde group. Phenylacetylene, with electron-withdrawing groups on its aromatic ring, afforded lower yields, due to the fact that this group reduces its nucleophilic activity (Table 2, entries 7–10). Because it is hard to form a Cu-alkyne intermediate with aliphatic alkyne, a trace product was observed when using aliphatic alkyne as a substrate (Table 2, entry 11). As the imine intermediate formed, aniline afforded no desired product (Table 2, entry 15). Unfortunately, aliphatic aldehydes afforded dissatisfactory yields (Table 2, entries 16–17), due to their low reactivity.

**Scheme 3:**
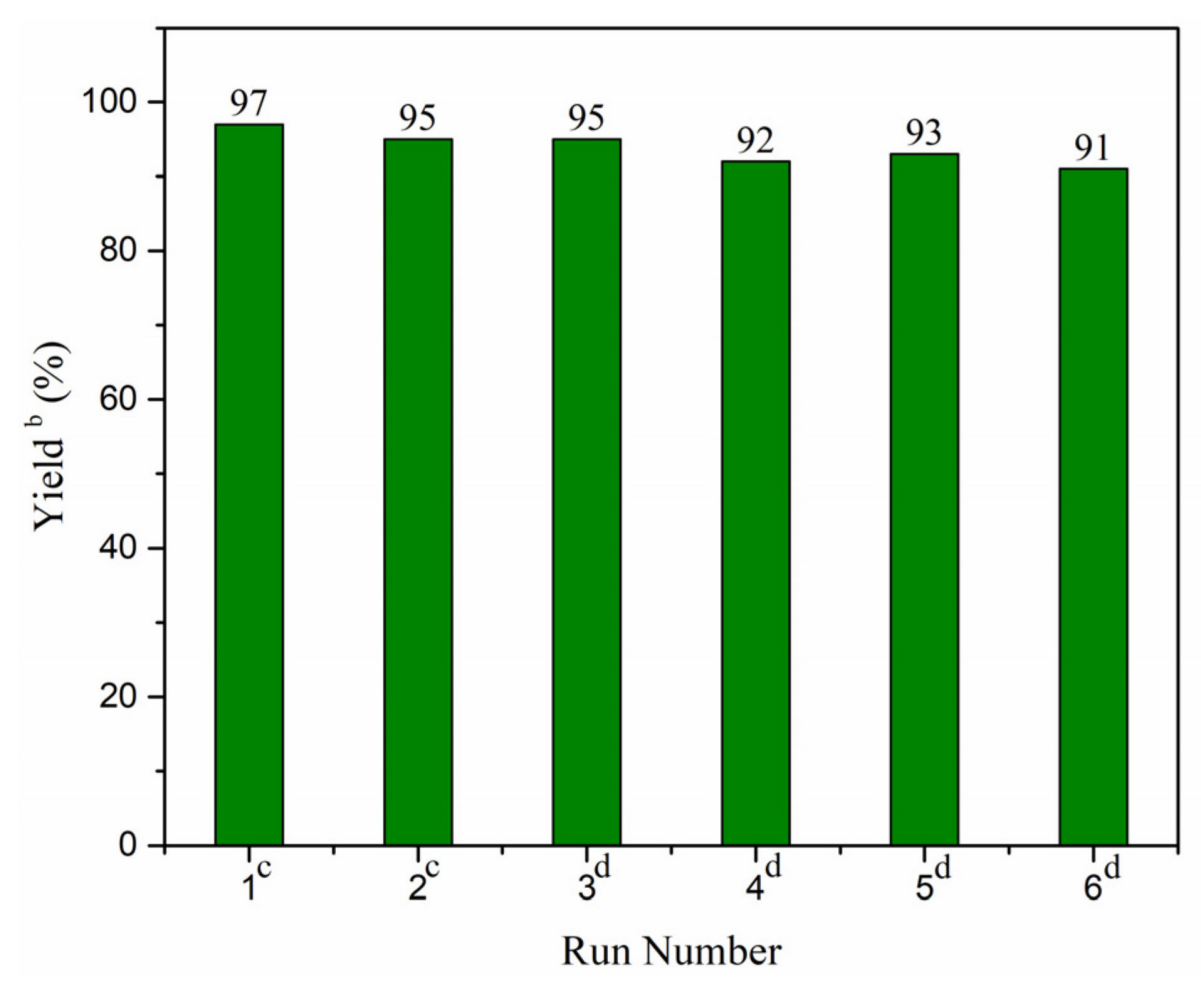
Reusability of Cu^II/I^@CMC-PANI dip catalyst in model A^3^ reaction ^a^. ^a^ Reaction conditions: *p*-chlorobenzaldehyde (1.0 mmol), morpholine (1.2 mmol), phenylacetylene (1.5 mmol), Cu^II/I^@CMC-PANI dip catalyst (2.7 mol% of Cu), toluene (2 mL), at 110 °C. ^b^ Isolated yields. ^c^ Reacted for 6 h. ^d^ Reacted for 10 h.

Furthermore, the recyclability and reusability of the Cu^II/I^@CMC-PANI film for the A^3^ model reaction was assessed. As shown in Scheme 3, the film could be recycled and reused successfully for up to six consecutive cycles, without significant losses in its activity. The Cu content, as measured by ICP-AES, in the recovered catalyst after two cycles was 1.705 mmol/g, which is slightly lower than that of 1.805 mmol/g in the fresh catalyst. Thus, to further investigate the Cu leaching of the catalyst in the A^3^ reaction, a hot filtration test was performed. The model A^3^ reaction was carried out at 110 °C for 1 h (36% conversion), and then the dip catalyst was picked up. The remaining mixture was further stirred for 5 h without the catalyst and a slight increase in conversation (36% to 50%) was observed, indeed suggesting that there was a small amount of Cu species leached out from the catalyst into the reaction mixture, which is consistent with the result of the ICP-AES analysis.

## 4. Conclusions

A novel and practical method for the fabrication of a Cu^II/I^@CMC-PANI film dip catalyst was developed via spray-assisted interfacial polymerization. In the preparation process, carboxymethylcellulose and copper sulfate served as the soft template and initiator, respectively, for the polymerization of aniline monomers, and the spraying method provided a unique interfacial layer to facilitate polymerization. This as-prepared film catalyst was well characterized by ICP, FT-IR, XPS, XRD, SEM, EDS, and TGA techniques. These characterized results proved that aniline had successfully polymerized to PANI onto the CMC molecular chain, and that Cu(II)/Cu(I) oxides, at the nano scale, were homogeneously loaded into the CMC-PANI film at the same time. The obverse and back side of the resultant film had different morphologies, due to their different PANI contents. In terms of application, the as-synthesized Cu^II/I^@CMC-PANI film dip catalyst possessed good thermal stability up to 200 °C, and could be efficiently applied in the A^3^ coupling reaction, due to its excellent recyclability and tolerance of a broad scope of substrates.

## Figures and Tables

**Table 1:** Optimization of model A^3^ reactions catalyzed by Cu^II/I^@CMC-PANI dip catalyst ^a^.

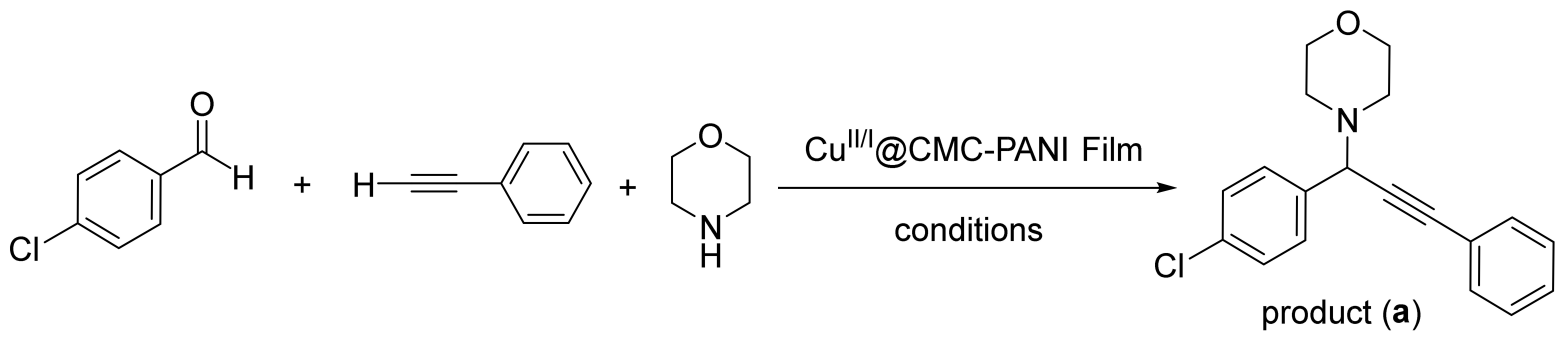					
**Entry**	Solvent	Dose of Catalyst (Mol% of Cu)	Temp. (°C)	Time (h)	Yield ^b^ (%)
1	H_2_O	2.7	100	12	N.R. ^c^
2	EtOH	2.7	78	12	6
3	*n*-BuOH	2.7	110	8	55
4	DMSO	2.7	110	8	38
5	DMF	2.7	110	8	51
6	CH_3_CN	2.7	80	12	trace ^d^
7	toluene	2.7	110	6	97
8	toluene	0	110	8	trace ^d^
9	toluene	1.8	110	8	93
10	toluene	3.6	110	8	97
11	toluene	2.7	90	8	12
12	toluene	2.7	70	24	trace ^d^

^a^ Reaction conditions: *p*-chlorobenzaldehyde (1.0 mmol), morpholine (1.2 mmol), phenylacetylene (1.5 mmol) and solvent (2 mL). ^b^ Isolated yields. ^c^ No reaction. ^d^ Observed by TLC.

**Table 2:** A^3^ coupling reactions catalyzed by Cu^II/I^@CMC-PANI film to synthesize a variety of propargylamines ^a^.

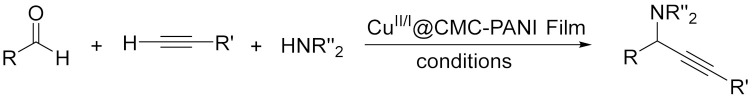					
Entry	R	R’	HNR’’_2_	Time (h)	Yield ^b^ (%)
1	4-Cl-Ph	Ph	morpholine	6	97 (a)
2	Ph	Ph	morpholine	8	91 (b)
3	4-Me-Ph	Ph	morpholine	8	83 (c)
4	2-MeO-Ph	Ph	morpholine	8	97 (d)
5	2-OH-Ph	Ph	morpholine	48	39 (e)
6	3-NO_2_-Ph	Ph	morpholine	48	86 (f)
7	Ph	3-Me-Ph	morpholine	16	87 (g)
8	Ph	4-MeO-Ph	morpholine	32	97 (h)
9	Ph	4-CF_3_-Ph	morpholine	48	83 (i)
10	Ph	4-Cl-Ph	morpholine	24	88 (j)
11	Ph	*n*-hexyl	morpholine	48	trace (k) ^c^
12	Ph	Ph	piperidine	48	84 (l)
13	4-Cl-Ph	Ph	piperidine	48	86 (m)
14	4-Cl-Ph	Ph	diethylamine	48	58 (n)
15	Ph	Ph	aniline	48	N.P. (o) ^d^
16	Et	Ph	morpholine	8	54 (p)
17	*i*-Pr	Ph	morpholine	8	30 (q)

^a^ Reaction conditions: aldehyde (1.0 mmol), amine (1.2 mmol), alkyne (1.5 mmol), Cu^II/I^@CMC-PANI film (2.7 mol% of Cu), toluene (2 mL), at 110 °C. ^b^ All yields are isolated. ^c^ Observed by TLC. ^d^ No product, and generated imine was observed by GC-MS.

## Data Availability

Data are within the article and Supplementary Materials.

## Supplementary Materials

The following supporting information can be downloaded at: https://www.mdpi.com/article/10.3390/nano12101641/s1, The spectra data, ^1^H and ^13^C NMR for all products (**a**–**q**), FT-IR ^19^F NMR and HRMS for new product (**i**). References [48,49,50,51,52,53,54,55,56,57,58,59,60,61] are cited in the Supplementary Materials.
