# Supplementary material for: Spray-Assisted Interfacial Polymerization to Form CuII/I@CMC-PANI Film: An Efficient Dip Catalyst for A3 Reaction
Source: Nanomaterials (Basel). 2022 May 11;12(10):1641. doi: 10.3390/nano12101641 (PMC9146272; doi:10.3390/nano12101641)

nanomaterials

Article

Spray-Assisted Interfacial Polymerization to Form $Cu^{II/I}$@CMC-PANI Film: An Efficient Dip Catalyst for A^3 Reaction

Zhian Xu [1], Liang Xiao [1], Xuetao Fan [1], Dongtao Lin [1], Liting Ma [2], Guochao Nie [2,*] and Yiqun Li [1,*]

[1] Department of Chemistry, College of Chemistry and Materials Science, Jinan University, Guangzhou 511443, China; zhian_xu@outlook.com (Z.X.); liang_xi728@163.com (L.X.); lovefandou1018@gmail.com (X.F.); Dongtao_Lin@163.com (D.L.)
[2] Photoelectric Information Center, School of Physics and Telecom, Yulin Normal University, Yulin 537000, China; mikt13f@163.com
* Correspondence: bccu518@163.com (G.N.); tlyq@jnu.edu.cn (Y.L.)

Outline

1. Spectral data for products and refrences………………….……………………… ..P2

2. Copies of ^{1}H, ^{13}C NMR for products ………………………………………….……..…..P8

3. Copies of ^{19}F NMR for synthesized organofluoride..……..………………...……… ..P23

4. Copies of FT-IR and HRMS for new compound..………………………………….. ..P24

1. Spectral data for products

4-[1-(4-Chlorophenyl)-3-phenyl-2-propyn-1-yl]-morpholine (a) [1]

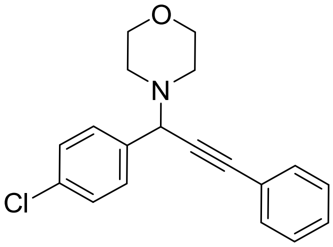

^{1}H NMR (300 MHz, chloroform-*d*) δ 7.55 (d, *J* = 8.4 Hz, 2H), 7.52 – 7.46 (m, 2H), 7.37 – 7.23 (m, 5H), 4.72 (s, 1H), 3.69 (q, *J* = 4.5 Hz, 4H), 2.58 (t, *J* = 4.7 Hz, 4H).
^{13}C NMR (75 MHz, chloroform-*d*) δ 136.51, 133.63, 131.90, 131.35, 129.98, 128.50, 128.45, 122.79, 89.03, 84.47, 67.10, 61.40, 49.84.

4-(1,3-Diphenyl-2-propyn-1-yl)-morpholine (b)[2]

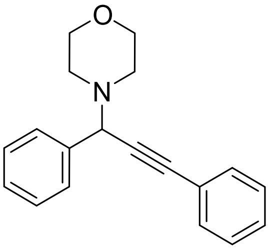

^{1}H NMR (300 MHz, chloroform-*d*) δ 7.62 (d, *J* = 6.7 Hz, 2H), 7.49 (dd, *J* = 6.8, 3.0 Hz, 2H), 7.41 – 7.19 (m, 6H), 4.75 (s, 1H), 3.77 – 3.59 (m, 4H), 2.68 – 2.50 (m, 4H).
^{13}C NMR (75 MHz, chloroform-*d*) δ 137.93, 131.93, 128.70, 128.45, 128.38, 128.36, 127.91, 123.09, 88.67, 85.22, 67.22, 62.12, 49.98.

4-[1-(4-Methylphenyl)-3-phenyl-2-propyn-1-yl]-morpholine (c)[3]

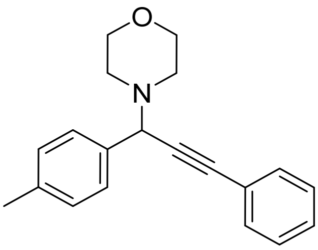

^{1}H NMR (300 MHz, chloroform-*d*) δ 7.55 – 7.42 (m, 4H), 7.34 – 7.20 (m, 3H), 7.14 (d, *J* = 7.9 Hz, 2H), 4.71 (s, 1H), 3.77 – 3.60 (m, 4H), 2.67 – 2.51 (m, 4H), 2.31 (s, 3H).
^{13}C NMR (75 MHz, chloroform-*d*) δ 137.50, 134.96, 131.92, 129.05, 128.64, 128.43, 128.32, 123.18, 88.43, 85.51, 67.24, 61.90, 50.00, 21.25.

4-[1-(2-Methoxyphenyl)-3-phenyl-2-propyn-1-yl]-morpholine (d)[4]

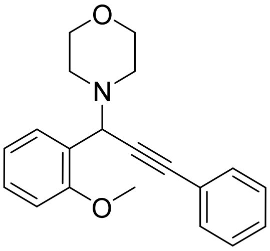

^{1}H NMR (300 MHz, chloroform-*d*) δ 8.64 (dd, *J* = 7.5, 1.8 Hz, 1H), 8.45 (dd, *J* = 6.6, 3.2 Hz, 2H), 8.23 (qt, *J* = 4.1, 1.8 Hz, 4H), 7.95 (td, *J* = 7.5, 1.1 Hz, 1H), 7.83 (dd, *J* = 8.3, 1.1 Hz, 1H), 6.19 (s, 1H), 4.75 (s, 3H), 4.73 – 4.58 (m, 4H), 3.77 – 3.55 (m, 4H).
^{13}C NMR (75 MHz, chloroform-*d*) δ 157.31, 131.85, 130.31, 129.26, 128.36, 128.19, 126.14, 123.30, 120.34, 111.32, 86.90, 86.74, 67.17, 55.92, 55.14, 50.25.

2-[1-(4-Morpholinyl)-3-phenyl-2-propyn-1-yl]-phenol (e)[5]

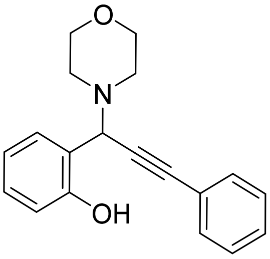

^{1}H NMR (300 MHz, chloroform-*d*) δ 11.76 (s, 1H), 8.55 (td, *J* = 7.5, 2.5 Hz, 3H), 8.39 – 8.28 (m, 3H), 8.23 (td, *J* = 7.7, 1.7 Hz, 1H), 7.93 – 7.82 (m, 2H), 6.07 (s, 1H), 4.86 – 4.67 (m, 4H), 3.75 (t, *J* = 4.9 Hz, 4H).
^{13}C NMR (75 MHz, chloroform-*d*) δ 157.14, 131.97, 129.85, 128.85, 128.54, 122.34, 120.65, 119.53, 116.60, 90.45, 81.80, 66.89, 60.75, 53.60.

4-[1-(3-Nitrophenyl)-3-phenyl-2-propyn-1-yl]-morpholine (f)[6]

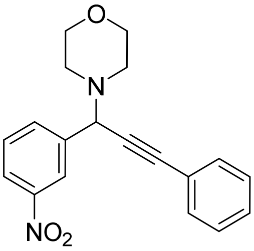

^{1}H NMR (300 MHz, chloroform-*d*) δ 8.54 (d, *J* = 2.1 Hz, 1H), 8.15 (dd, *J* = 8.3, 2.3 Hz, 1H), 8.01 (d, *J* = 8.6 Hz, 1H), 7.63 – 7.47 (m, 3H), 7.40 – 7.27 (m, 3H), 4.88 (s, 1H), 3.83 – 3.65 (m, 4H), 2.63 (ddt, *J* = 16.4, 11.9, 6.7 Hz, 4H).
^{13}C NMR (75 MHz, chloroform-*d*) δ 148.38, 140.44, 134.55, 131.90, 129.22, 128.71, 128.47, 123.46, 122.90, 122.37, 89.82, 83.29, 67.01, 61.30, 49.80.

4-[3-(3-methylphenyl)-1-phenyl-2-propyn-1-yl]-morpholine (g)[7]

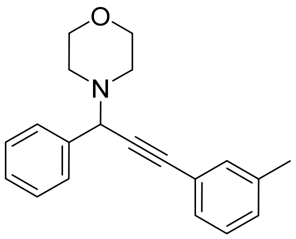

^{1}H NMR (300 MHz, chloroform-*d*) δ 7.62 (dd, *J* = 7.2, 1.8 Hz, 2H), 7.39 – 7.23 (m, 5H), 7.17 (t, *J* = 7.5 Hz, 1H), 7.07 (d, *J* = 7.7 Hz, 1H), 4.75 (s, 1H), 3.78 – 3.60 (m, 4H), 2.69 – 2.51 (m, 4H), 2.29 (s, 3H).
^{13}C NMR (75 MHz, chloroform-*d*) δ 138.07, 138.01, 132.53, 129.27, 129.01, 128.70, 128.37, 128.35, 127.88, 122.92, 88.88, 84.78, 67.23, 62.13, 49.99, 21.33.

4-[3-(4-methoxyphenyl)-1-phenyl-2-propyn-1-yl]-morpholine (h)[8]

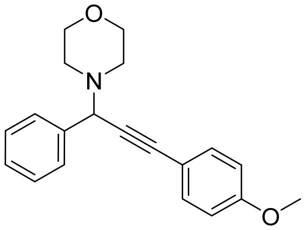

^{1}H NMR (300 MHz, chloroform-*d*) δ 7.66 – 7.57 (m, 2H), 7.47 – 7.39 (m, 2H), 7.38 – 7.22 (m, 3H), 6.86 – 6.77 (m, 2H), 4.74 (s, 1H), 3.73 (s, 3H), 3.69 (dq, *J* = 5.3, 3.1 Hz, 4H), 2.59 (dq, *J* = 5.2, 2.8 Hz, 4H).
^{13}C NMR (75 MHz, chloroform-*d*) δ 159.67, 138.14, 133.28, 128.67, 128.28, 127.79, 115.16, 114.02, 88.43, 83.64, 67.21, 62.13, 55.28, 49.96.

4-[3-[4-(trifluoromethyl)-1-phenyl-2-propyn-1-yl]-2-propyn-1-yl]-morpholine (i)

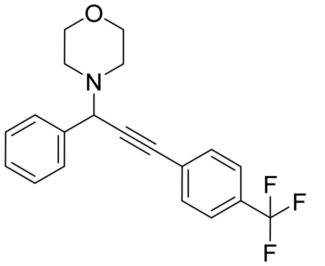

^{1}H NMR (300 MHz, chloroform-*d*) δ 7.70 – 7.53 (m, 6H), 7.41 – 7.26 (m, 3H), 4.80 (s, 1H), 3.81 – 3.64 (dq, 4H), 2.63 (dq, *J* = 5.2, 2.6 Hz, 4H).
^{13}C NMR (75 MHz, chloroform-*d*) δ 137.34, 132.11, 130.28, 129.85, 128.57, 128.38, 128.02, 126.81, 126.79, 125.37, 125.32, 125.27, 125.22, 87.90, 87.27, 67.09, 62.07, 49.95.
^{19}F NMR (282 MHz, Chloroform-*d*) δ -62.71.
FTIR ν: 2855, 1615, 1451, 1405, 1321, 1288, 1274, 1248, 1166, 1115, 1106, 1066, 1030, 1018, 1003, 983, 971, 936, 916, 865, 842, 799, 750, 722, 698, 644, 597, 582, 550.
HRMS (ESI-TOF) m/z: Calcd for $C_{20}H_{19}F_3NO^+$ [M+H$^+$] 346.1413, found 346.1417.

4-[3-(4-chlorophenyl)-1-phenyl-2-propyn-1-yl]-morpholine (j)[9]

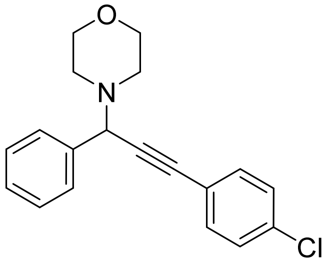

^{1}H NMR (300 MHz, chloroform-*d*) δ 7.65 – 7.55 (m, 2H), 7.48 – 7.24 (m, 7H), 4.77 (s, 1H), 3.81 – 3.63 (m, 4H), 2.70 – 2.53 (m, 4H).
^{13}C NMR (75 MHz, chloroform-*d*) δ 137.58, 134.31, 133.07, 128.69, 128.57, 128.32, 127.91, 121.46, 87.40, 86.21, 67.15, 62.07, 49.94.

1-(1,3-diphenyl-2-propyn-1-yl)-piperidine (l)[10]

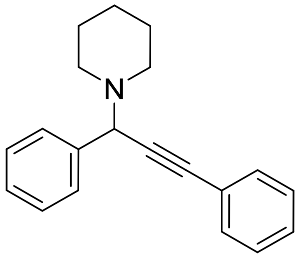

^{1}H NMR (300 MHz, chloroform-*d*) δ 7.66 – 7.60 (m, 2H), 7.54 – 7.49 (m, 2H), 7.38 – 7.28 (m, 6H), 4.83 (s, 1H), 2.58 (t, *J* = 5.4 Hz, 4H), 1.60 (h, *J* = 5.1 Hz, 4H), 1.45 (q, *J* = 5.7 Hz, 2H).
^{13}C NMR (75 MHz, chloroform-*d*) δ 138.41, 131.84, 129.00, 128.64, 128.31, 128.10, 127.54, 123.34, 87.93, 86.00, 62.35, 50.67, 26.11, 24.42.

1-[1-(4-Chlorophenyl)-3-phenyl-2-propyn-1-yl]piperidine (m)[11]

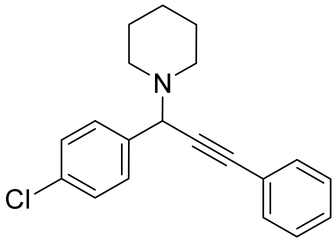

^{1}H NMR (300 MHz, chloroform-*d*) δ 7.61 – 7.55 (m, 2H), 7.50 (dq, *J* = 7.0, 2.1 Hz, 2H), 7.32 (ddd, *J* = 7.0, 4.0, 1.9 Hz, 5H), 4.87 (s, 1H), 2.60 (dt, *J* = 6.5, 3.6 Hz, 4H), 1.61 (h, *J* = 5.5 Hz, 4H), 1.45 (q, *J* = 5.8 Hz, 2H).
^{13}C NMR (75 MHz, chloroform-*d*) δ 136.55, 133.49, 131.84, 130.10, 128.37, 128.36, 128.28, 122.93, 88.48, 84.98, 61.55, 50.49, 25.82, 24.23.

4-Chloro-N, N-diethyl-α-(2-phenylethynyl)benzenemethanamine (n)[12]

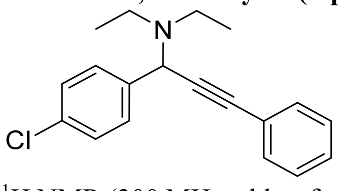

^{1}H NMR (300 MHz, chloroform-*d*) δ 7.65 – 7.61 (m, 2H), 7.52 – 7.48 (m, 2H), 7.35 – 7.29 (m, 5H), 5.02 (s, 1H), 2.72 – 2.46 (m, 4H), 1.08 (t, *J* = 7.1 Hz, 6H).
^{13}C NMR (75 MHz, chloroform-*d*) δ 138.35, 133.07, 131.82, 129.79, 128.36, 128.24, 128.20, 123.11, 87.91, 85.37, 56.52, 44.63, 13.48.

4-(1-Ethyl-3-phenyl-2-propyn-1-yl)morpholine (p)[13]

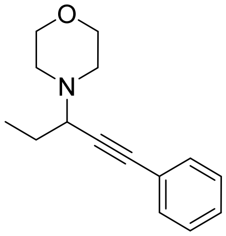

^{1}H NMR (300 MHz, chloroform-*d*) δ 7.47 – 7.39 (m, 2H), 7.32 – 7.24 (m, 3H), 3.83 – 3.65 (m, 4H), 3.40 (t, *J* = 7.5 Hz, 1H), 2.82 – 2.63 (m, 2H), 2.62 – 2.49 (m, 2H), 1.73 (p, *J* = 7.4 Hz, 2H), 1.07 (t, *J* = 7.4 Hz, 3H).
^{13}C NMR (75 MHz, chloroform-*d*) δ 131.73, 128.26, 127.98, 123.24, 87.03, 86.26, 67.11, 59.80, 49.76, 26.11, 11.27.

4-[1-(1-Methylethyl)-3-phenyl-2-propyn-1-yl]morpholine (q)[14]

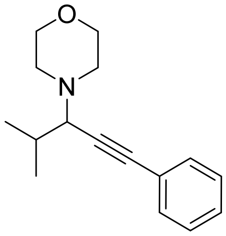

^{1}H NMR (300 MHz, Chloroform-*d*) δ 7.50 – 7.37 (m, 2H), 7.35 – 7.23 (m, 3H), 3.83 – 3.65 (m, 4H), 3.01 (d, *J* = 9.7 Hz, 1H), 2.76 – 2.63 (m, 2H), 2.58 – 2.44 (m, 2H), 1.89 (dh, *J* = 9.6, 6.6 Hz, 1H), 1.11 (d, *J* = 6.6 Hz, 3H), 1.03 (d, *J* = 6.6 Hz, 3H).

^{13}C NMR (75 MHz, Chloroform-*d*) δ 131.73, 128.26, 127.90, 123.42, 86.73, 86.65, 67.23, 65.23, 50.03, 29.94, 20.38, 19.83.

References

1. Kidwai, M.; Jahan, A. Nafion® NR50 catalyzed A^3-coupling for the synthesis of propargylamines via C-H activation. *J. Iran. Chem. Soc.* **2011**, *8*, 462-469.
2. Samai, S.; Nandi, G. C.; Singh, M. S. An efficient and facile one-pot synthesis of propargylamines by three-component coupling of aldehydes, amines, and alkynes via C–H activation catalyzed by $NiCl_2$. *Tetrahedron Lett.* **2010**, *51*, 5555-5558. DOI: 10.1016/j.tetlet.2010.08.043.
3. Zhu, W.; Qian, W.; Zhang, Y. Synthesis of 1, 3-diaryl-3-aminopropynes via the dethiolation of thioamides promoted by the samarium/samarium diiodide mixed reagent. *J. Chem. Res.* **2005**, *2005*, 410-412.
4. Ren, G.; Zhang, J.; Duan, Z.; Cui, M.; Wu, Y. A simple and economic synthesis of propargylamines by CuI-catalyzed three-component coupling reaction with succinic acid as additive. *Aust. j. Chem.* **2009**, *62*, 75-81.
5. Namitharan, K.; Pitchumani, K. Nickel-catalyzed solvent-free three-component coupling of aldehyde, alkyne and amine. *Eur. J. Org. Chem.* **2010**, *2010*, 411-415. DOI: 10.1002/ejoc.200901084.
6. Fodor, A.; Kiss, A.; Debreczeni, N.; Hell, Z.; Gresits, I. A simple method for the preparation of propargylamines using molecular sieve modified with copper(II). *Org. Biomol. Chem.* **2010**, *8*, 4575-4581. DOI: 10.1039/c0ob00224k.
7. Samanta, S.; Hajra, A. Divergent synthesis of allenylsulfonamide and enaminonesulfonamide via In(III)-catalyzed couplings of propargylamine and N-fluorobenzenesulfonimide. *J. Org. Chem.* **2018**, *83*, 13157-13165. DOI: 10.1021/acs.joc.8b01882.
8. Zhang, Q.; Chen, J.-X.; Gao, W.-X.; Ding, J.-C.; Wu, H.-Y. Copper-catalyzed one-pot synthesis of propargylamines via C-H activation in PEG. *Appl. Organomet. Chem.* **2010**, *24*, 809-812. DOI: 10.1002/aoc.1707.
9. Sun, R.; Liu, J.; Yang, S.; Chen, M.; Sun, N.; Chen, H.; Xie, X.; You, X.; Li, S.; Liu, Y. Cp_2TiCl_2-catalyzed cis-hydroalumination of propargylic amines with Red-Al: stereoselective synthesis of Z-configured allylic amines. *Chem. Commun.* **2015**, *51*, 6426-6429. DOI: 10.1039/c5cc00950b.
10. Munshi, A. M.; Agarwal, V.; Ho, D.; Raston, C. L.; Saunders, M.; Smith, N. M.; Iyer, K. S. Magnetically directed assembly of nanocrystals for catalytic control of a three-component coupling reaction. *Cryst. Growth Des.* **2016**, *16*, 4773-4776. DOI: 10.1021/acs.cgd.6b00582.
11. Wang, L.; Cai, C. Reusable polymer-anchored amino acid copper complex for the synthesis of propargylamines. *J. Chem. Res.* **2008**, *2008*, 538-541.
12. Zhou, Y.; He, T.; Wang, Z. Nanoparticles of silver oxide immobilized on different templates: highly efficient catalysts for three-component coupling of aldehyde-amine-alkyne. *Arkivoc* **2008**, *8*, 80-90.
13. Han, L.; Li, S. J.; Zhang, X. T.; Tian, S. K., Aromatic aza-claisen rearrangement of arylpropargylammonium salts generated in situ from arynes and tertiary propargylamines. *Chem.-Eur. J.* **2021,** *27*, 3091-3097.
14. Idzik, K.; Cabaj, J.; Sooducho, J.; Abdel-Fattah, A. A., Classical benzotriazole-mediatedα-aminoalkylations of alkynes: synthesis and characterization of alk-2-yn-1-amines as amphiphilic materials. *Helv. Chim. Acta* **2010,** *90*, 1672-1680.

2. Copies of ^1H, ^{13}C NMR for products

4-[1-(4-Chlorophenyl)-3-phenyl-2-propyn-1-yl]-morpholine (a)

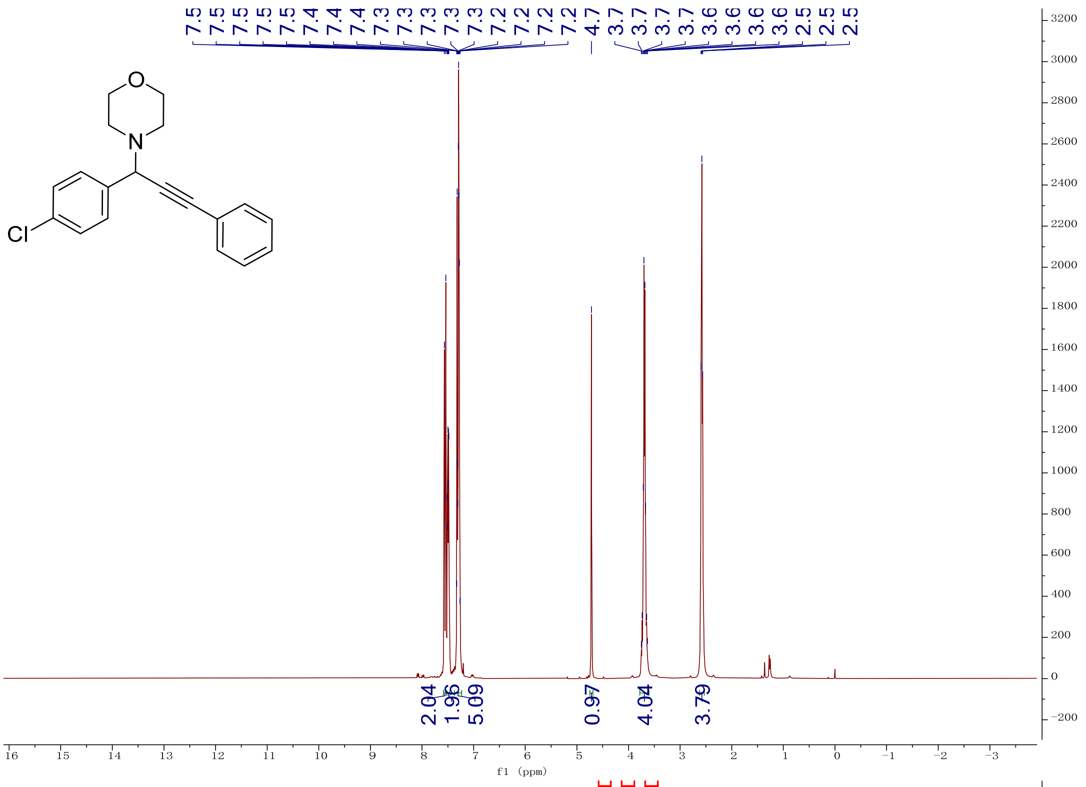

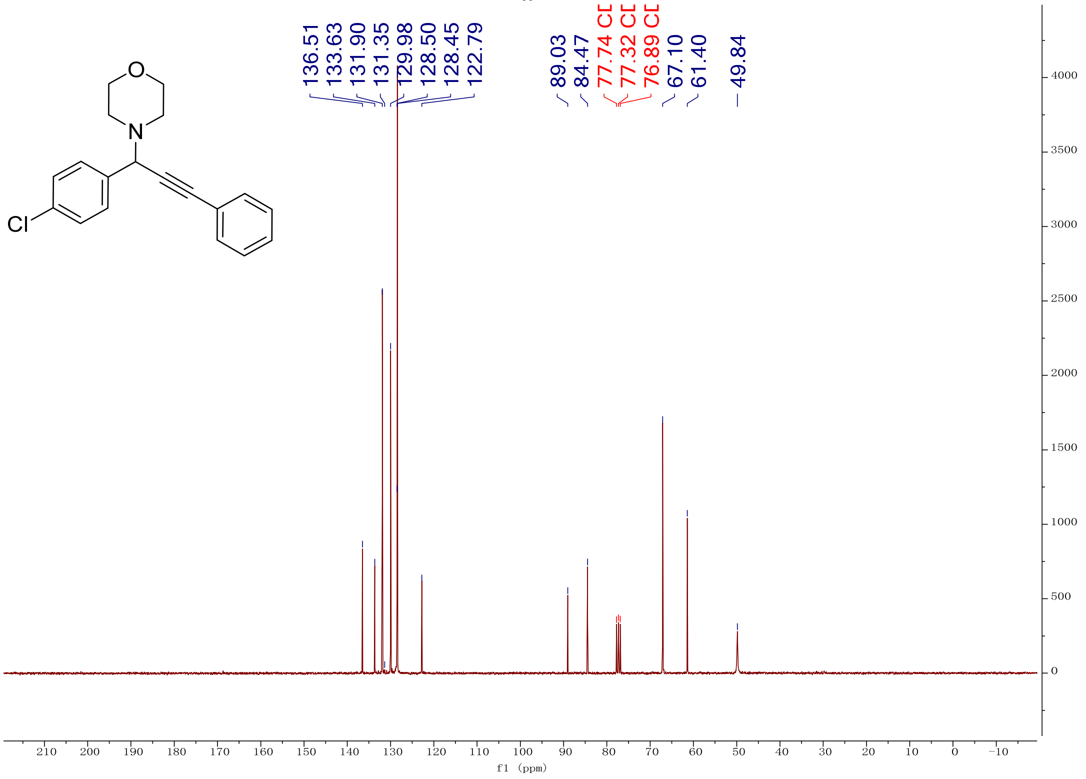

4-(1,3-Diphenyl-2-propyn-1-yl)-morpholine (b)

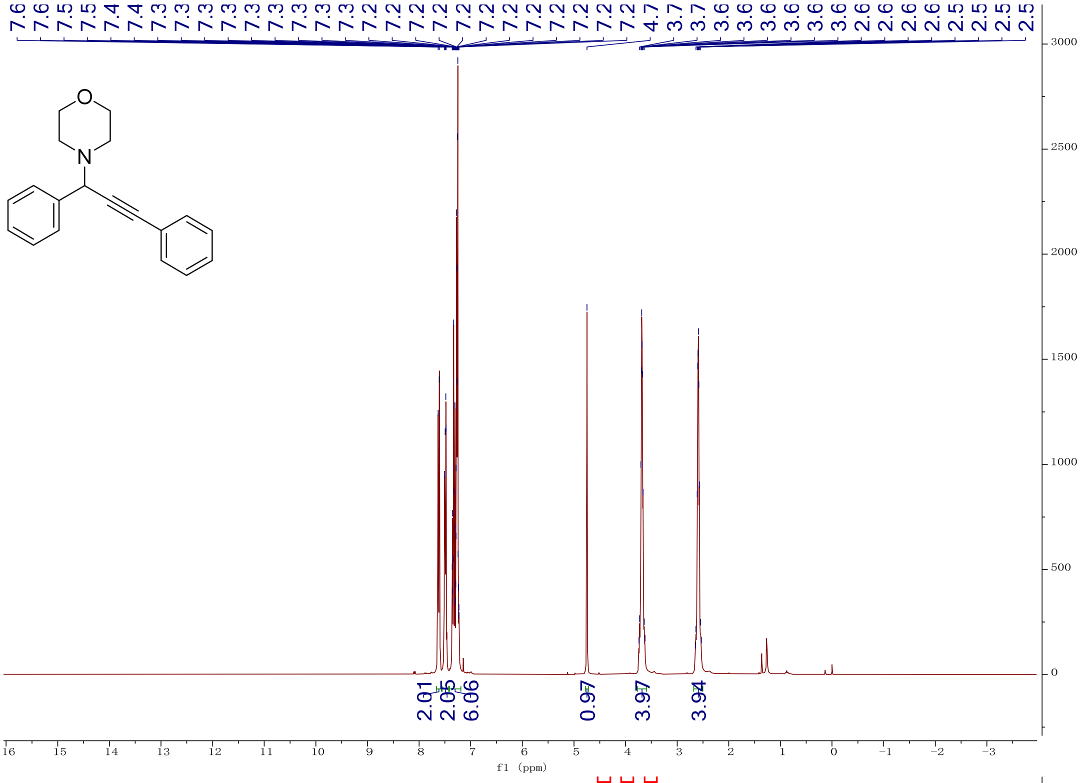

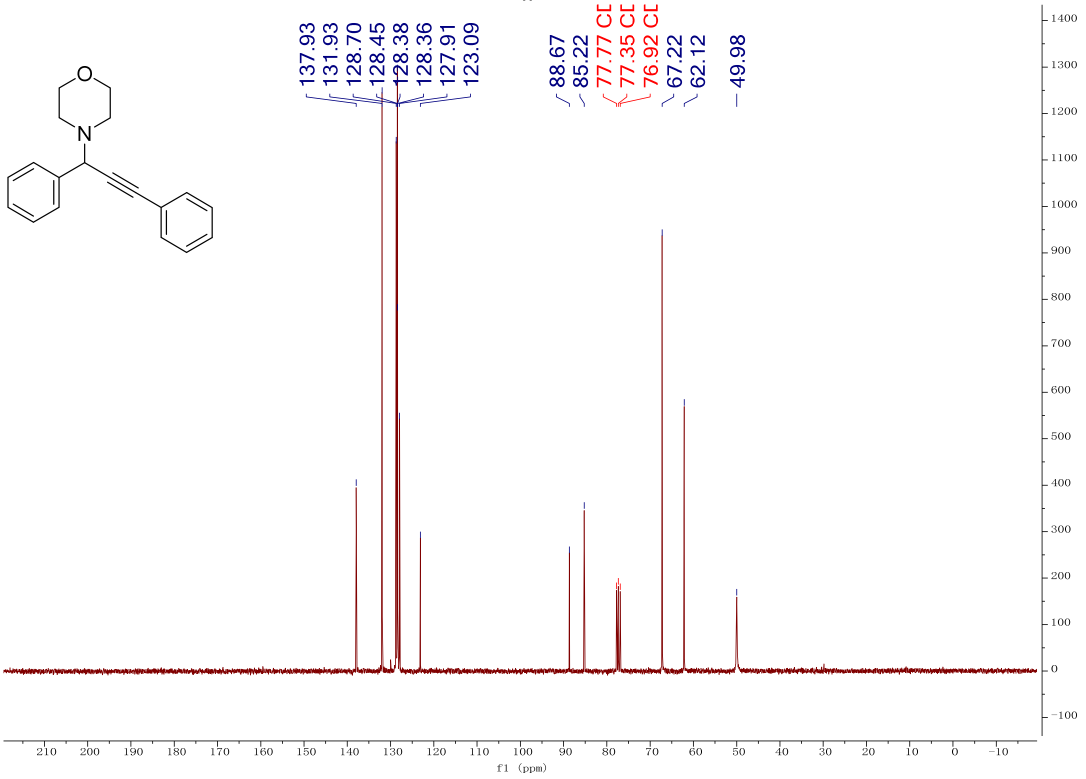

4-[1-(4-Methylphenyl)-3-phenyl-2-propyn-1-yl]-morpholine (c)

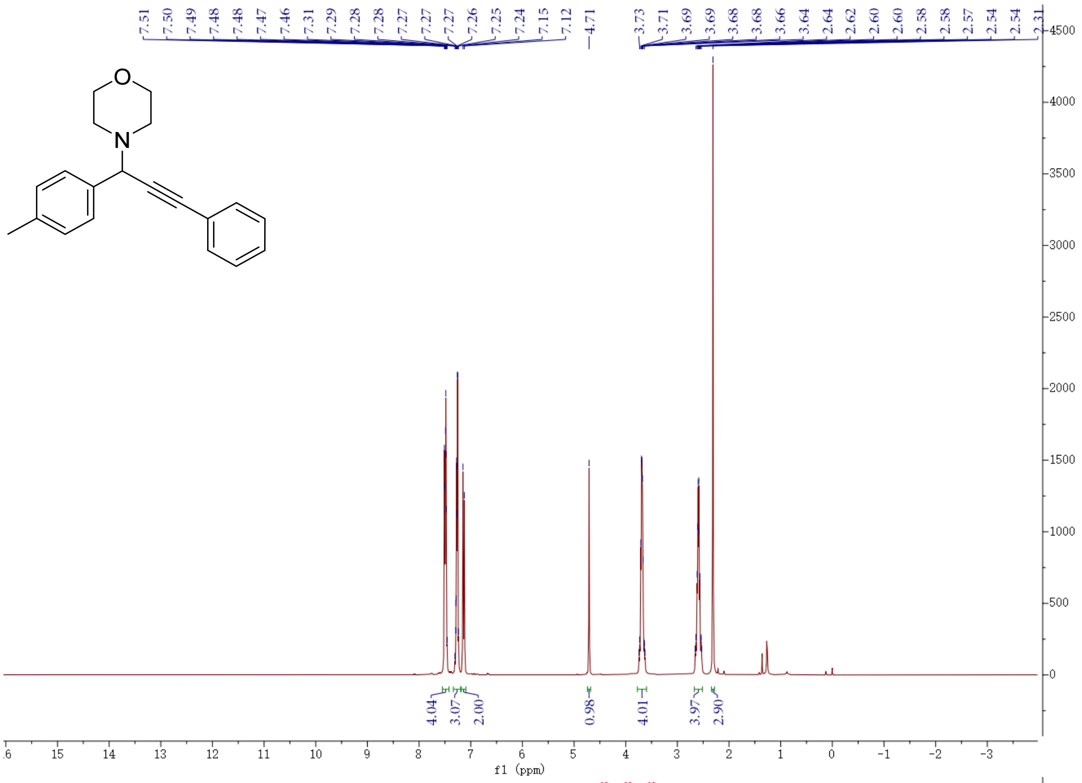

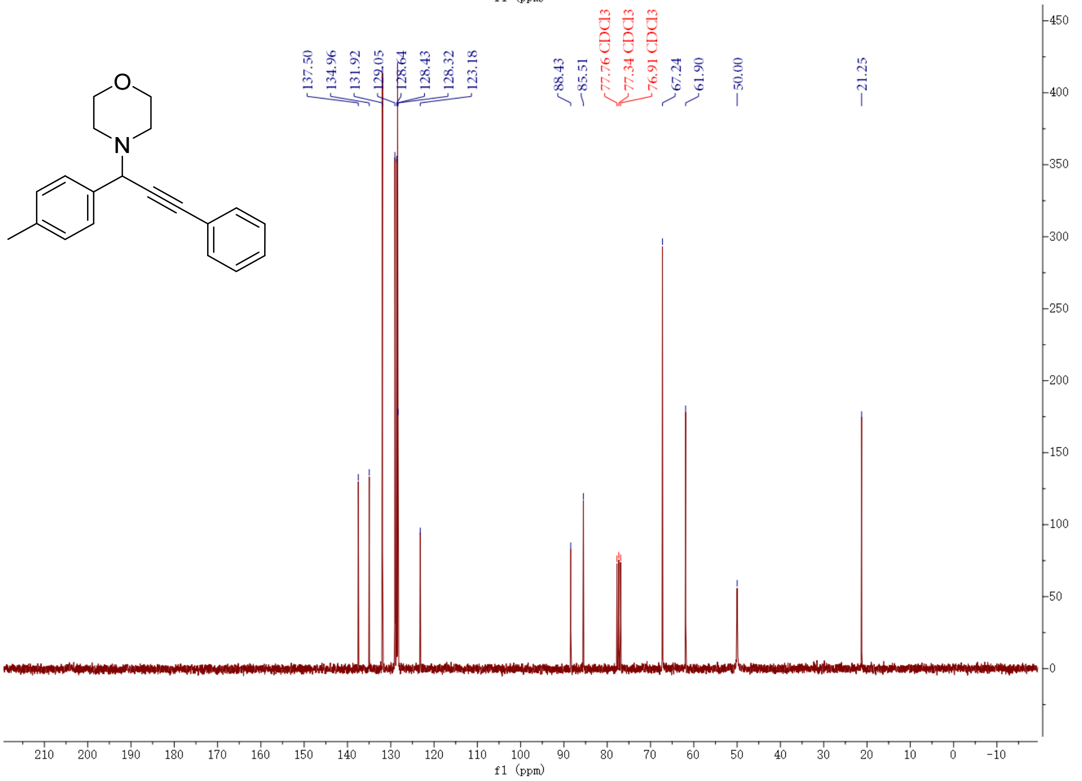

4-[1-(2-Methoxyphenyl)-3-phenyl-2-propyn-1-yl]-morpholine (d)

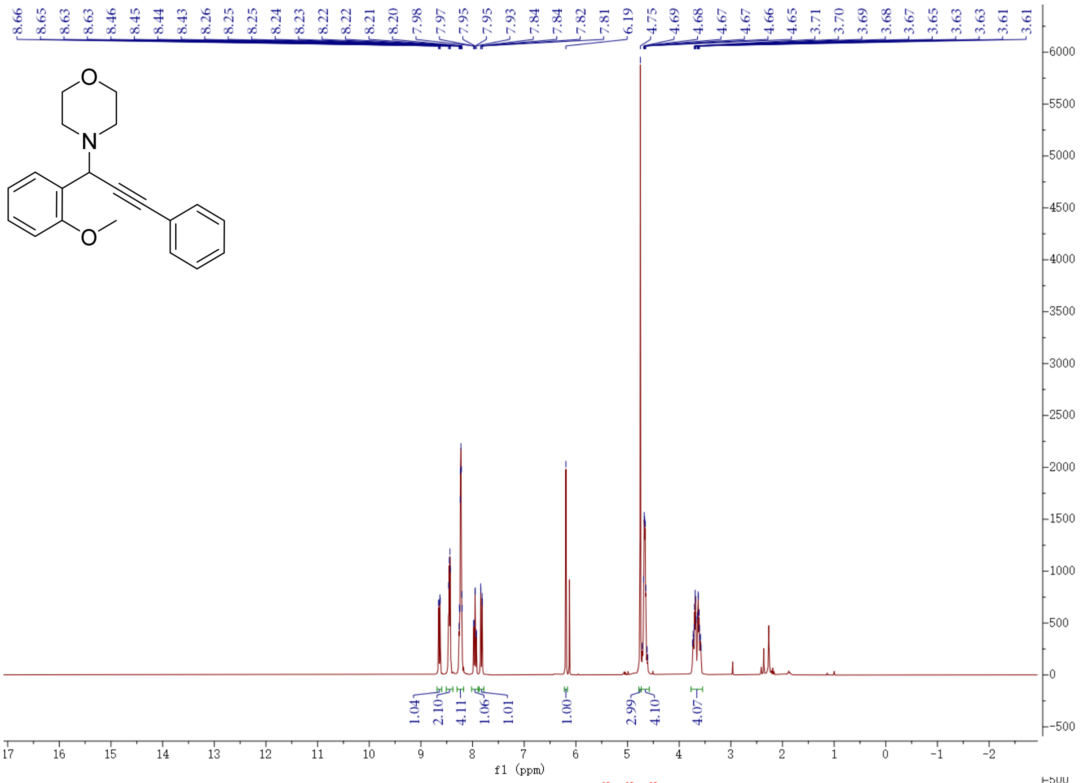

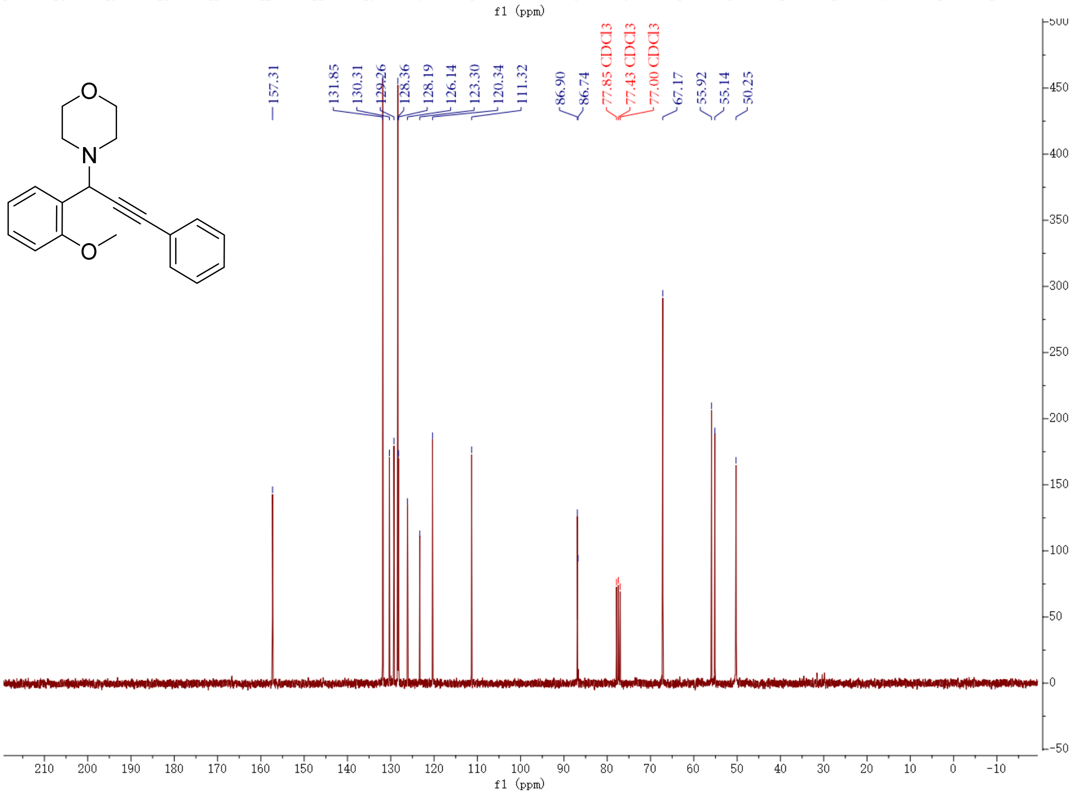

2-[1-(4-Morpholinyl)-3-phenyl-2-propyn-1-yl]-phenol (e)

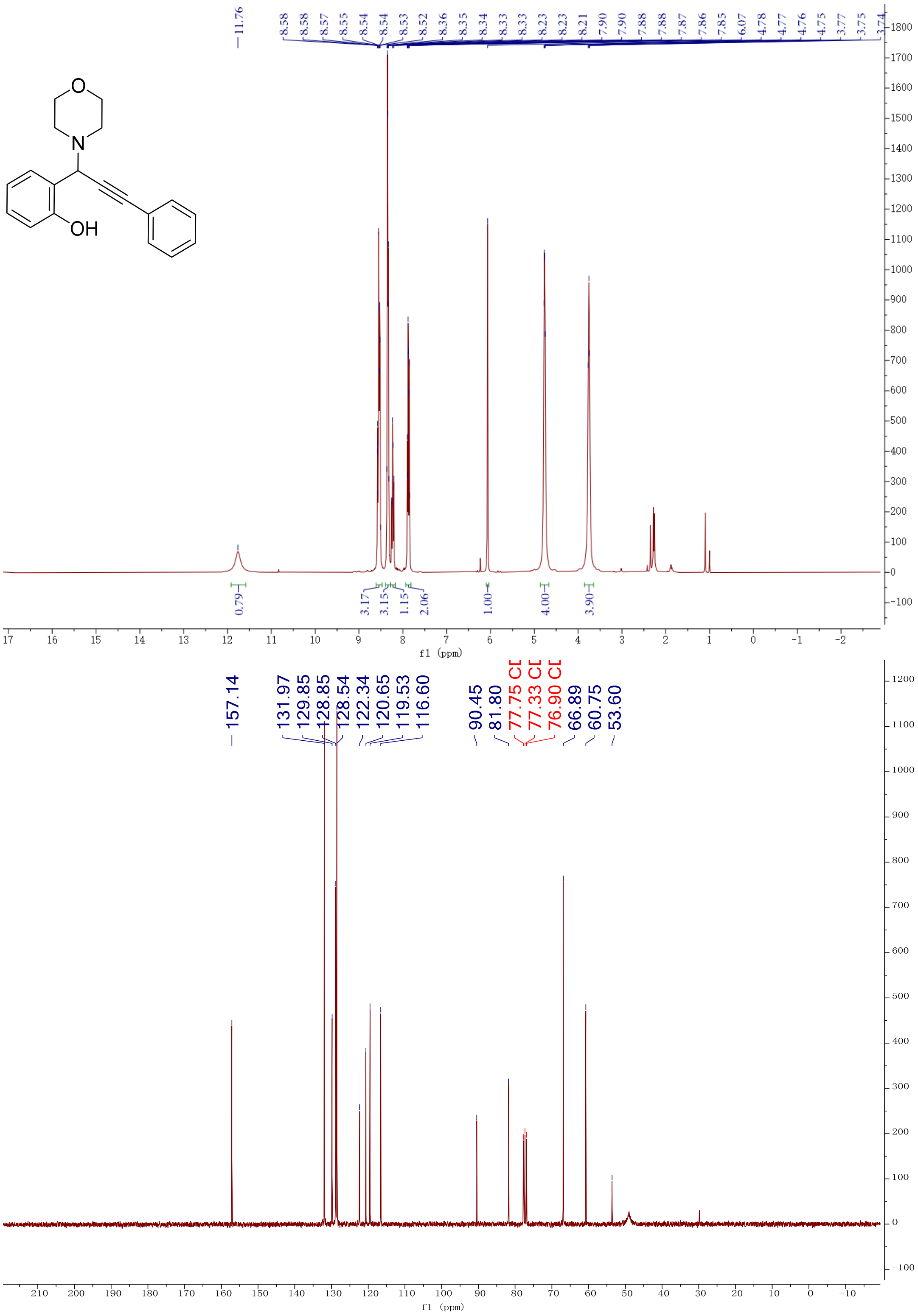

4-[1-(3-Nitrophenyl)-3-phenyl-2-propyn-1-yl]-morpholine (f)

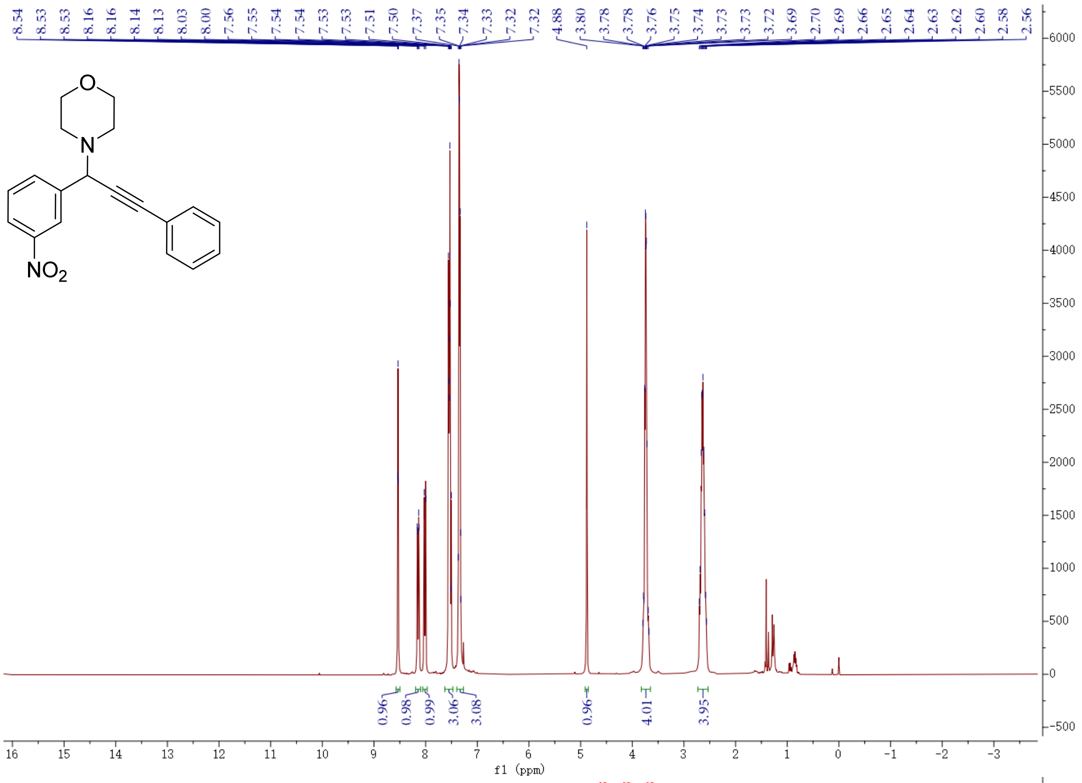

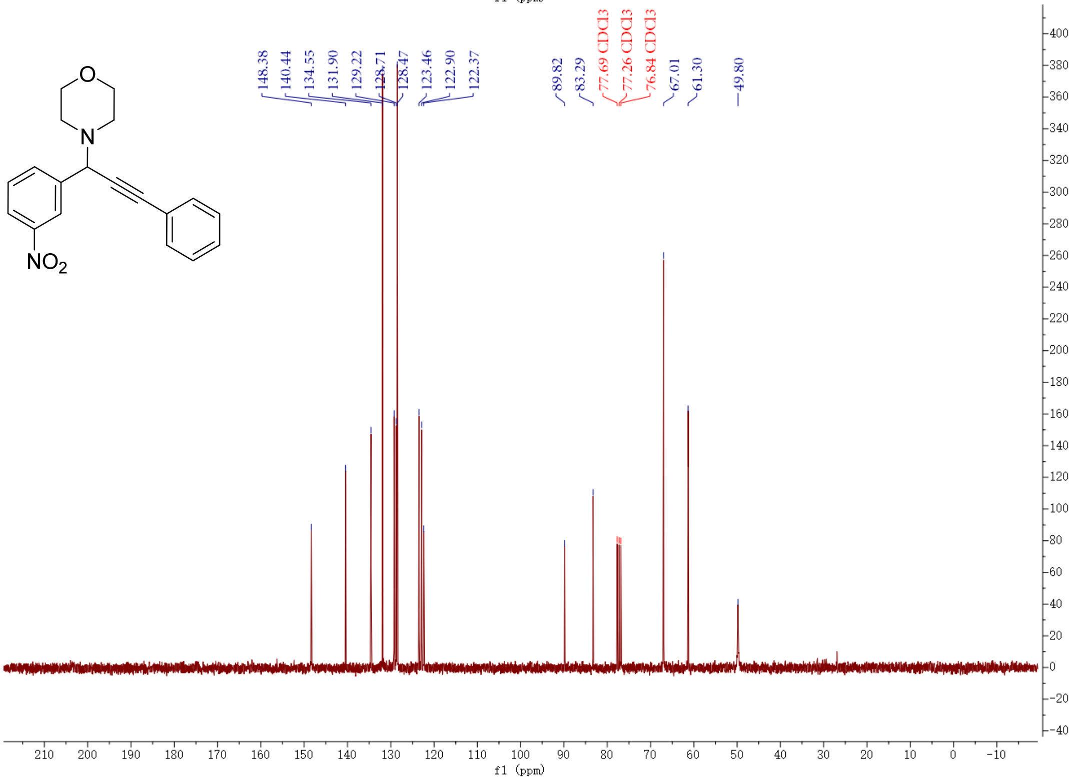

4-[3-(3-methylphenyl)-1-phenyl-2-propyn-1-yl]-morpholine (g)

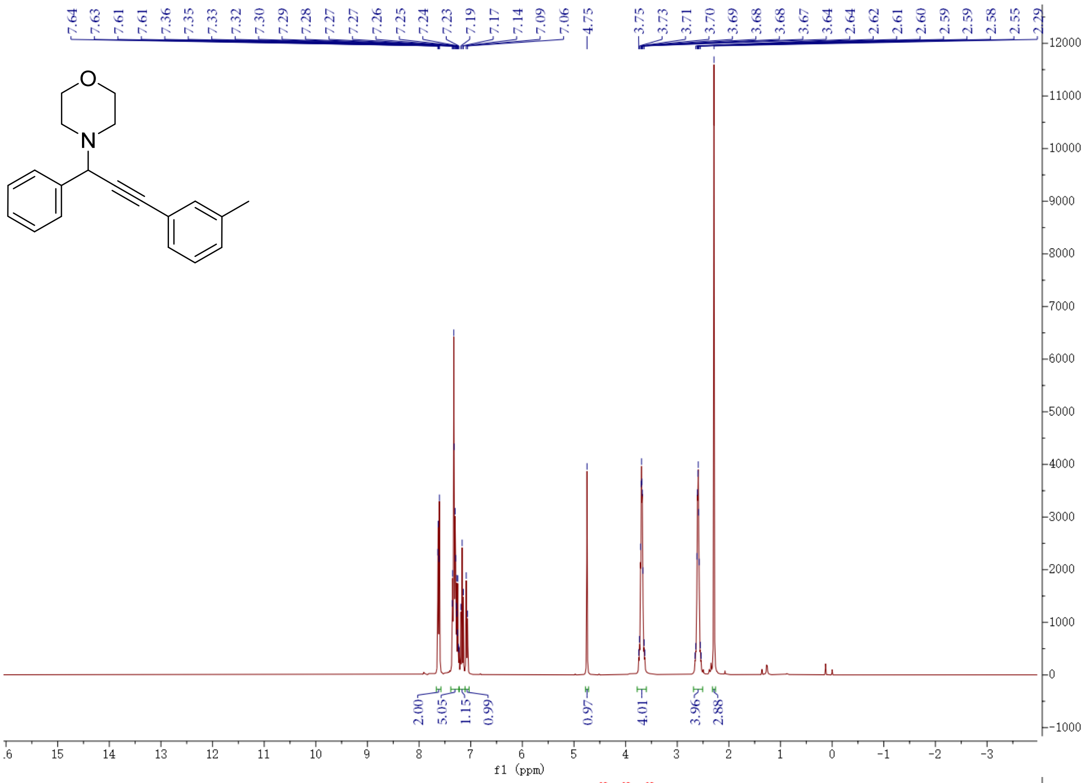

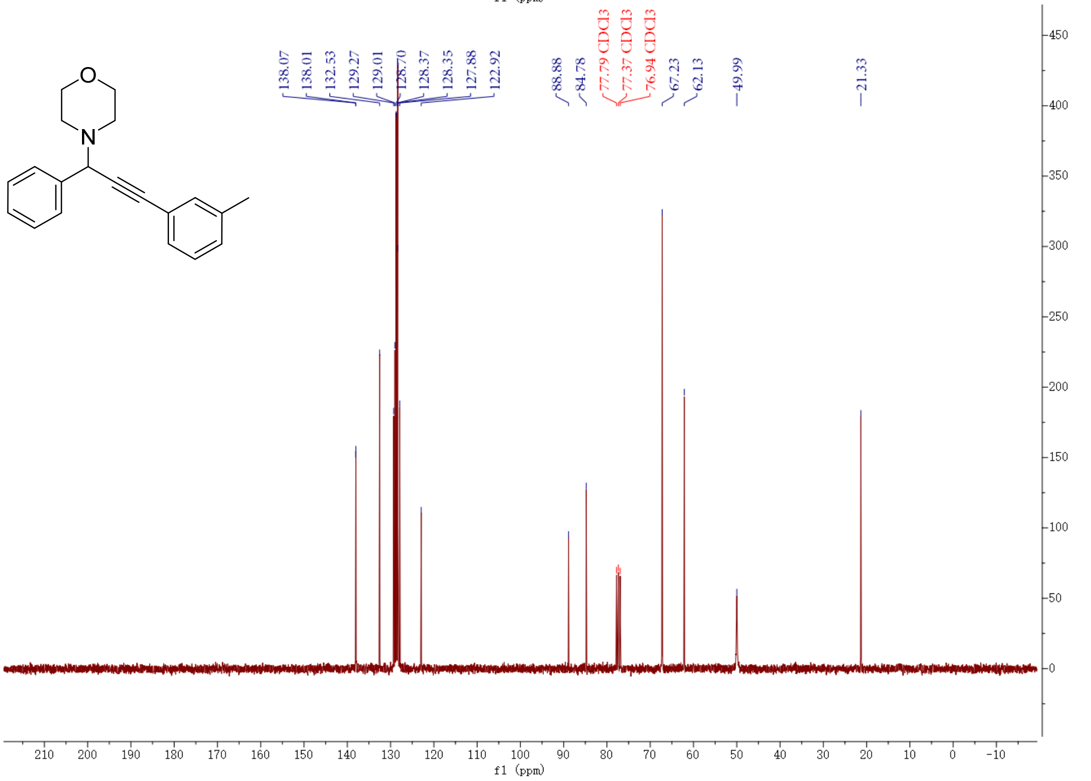

4-[3-(4-methoxyphenyl)-1-phenyl-2-propyn-1-yl]-morpholine (h)

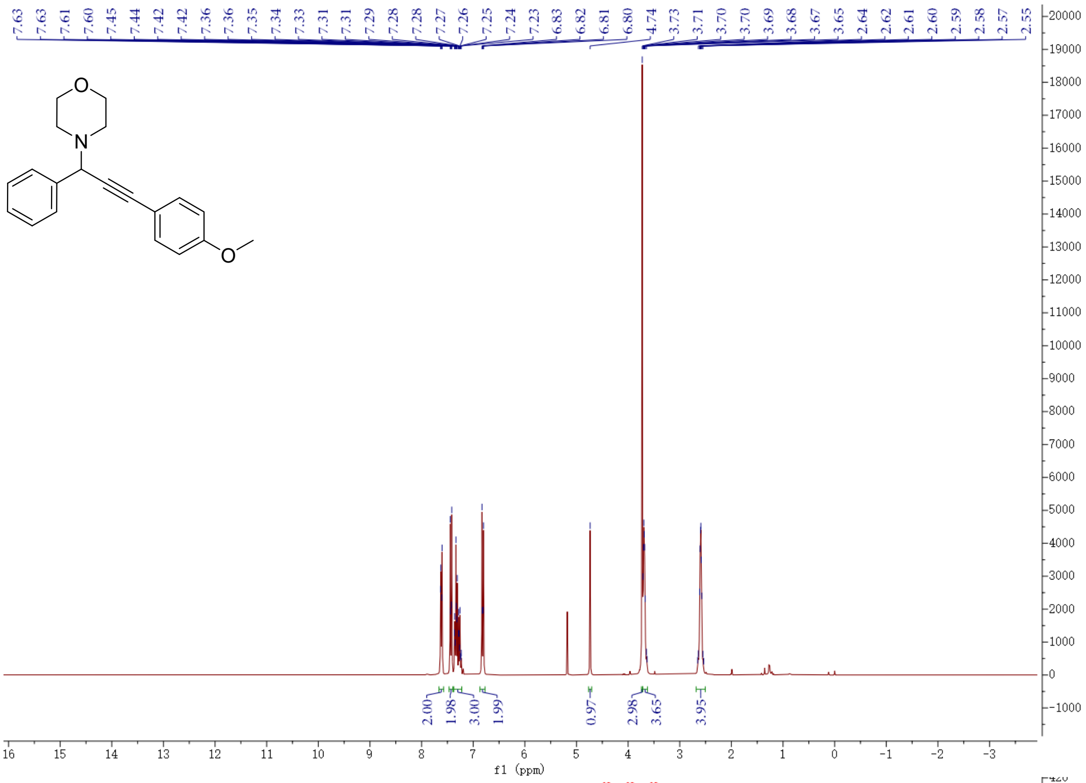

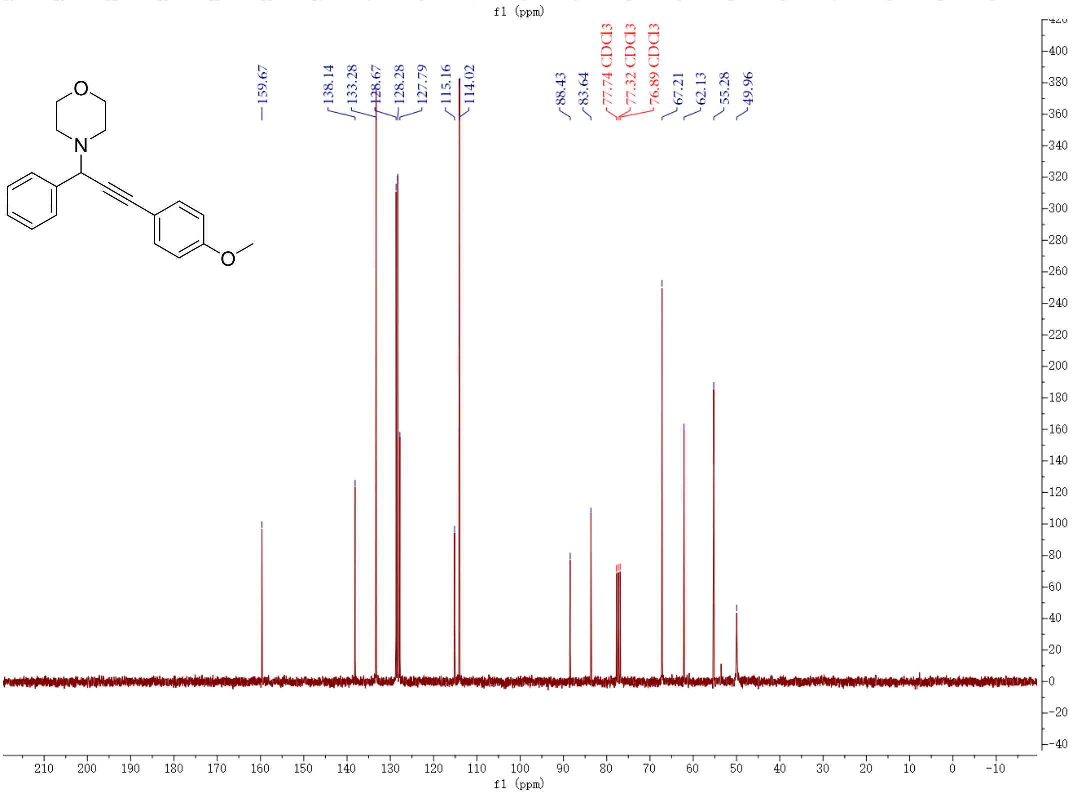

4-[3-[4-(trifluoromethyl)-1-phenyl-2-propyn-1-yl]-2-propyn-1-yl]-morpholine (i)

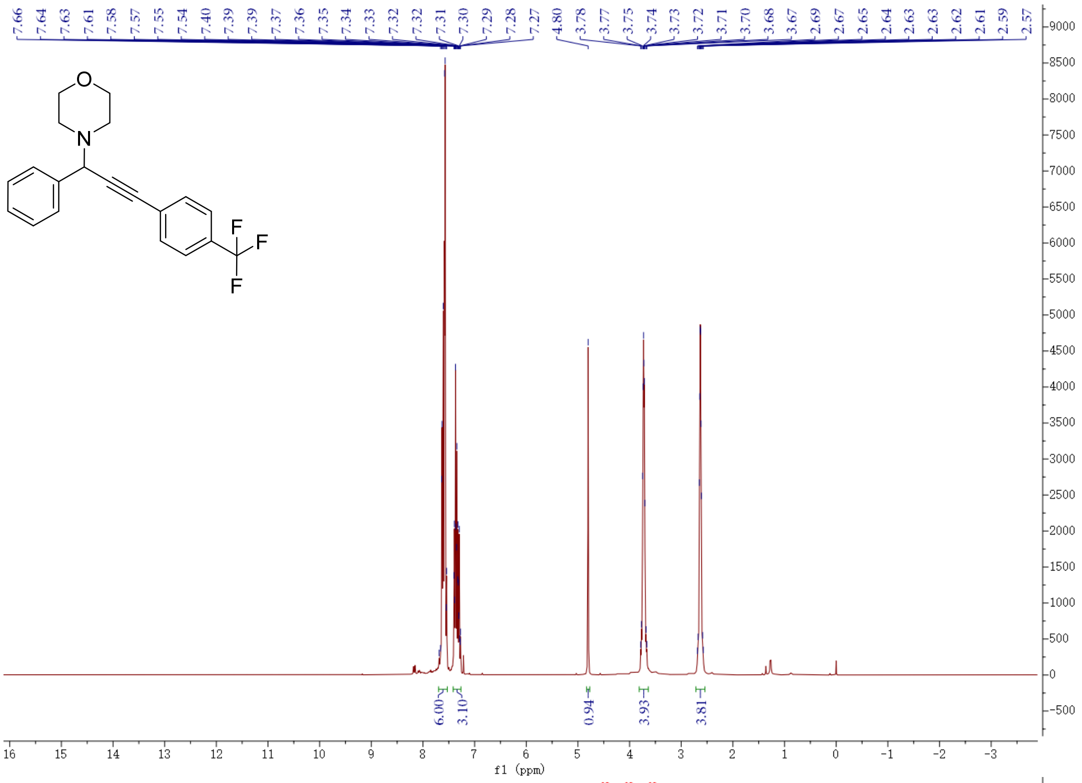

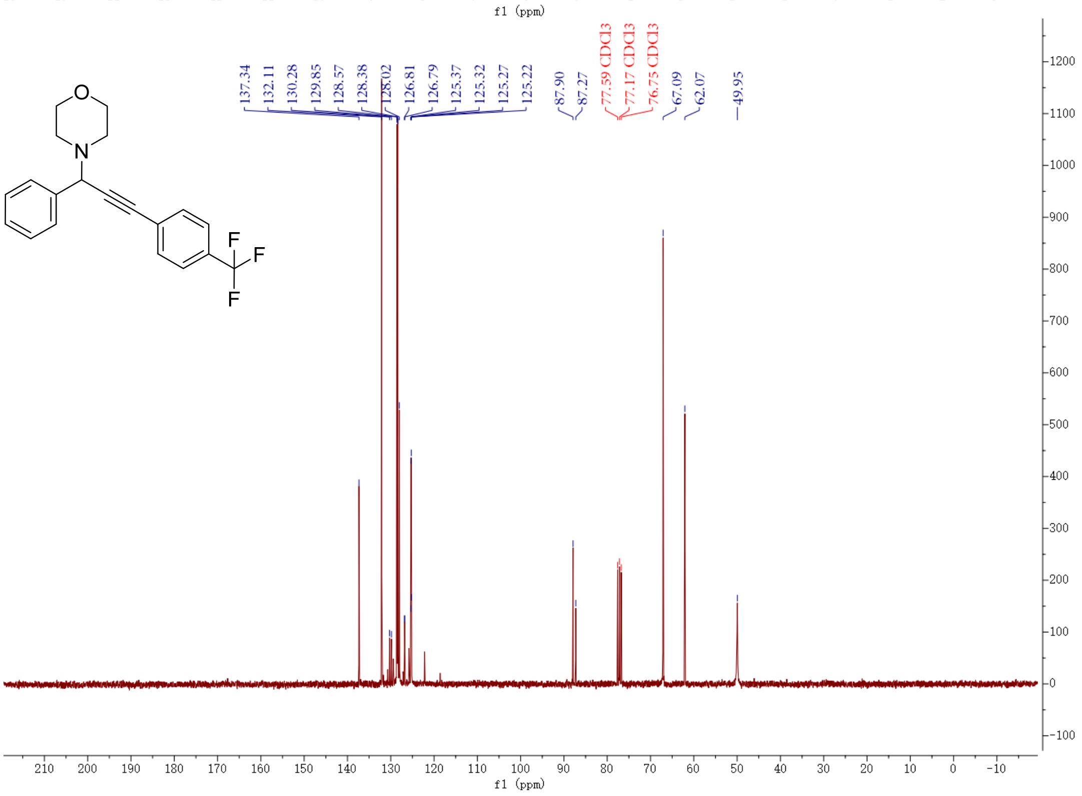

4-[3-(4-chlorophenyl)-1-phenyl-2-propyn-1-yl]-morpholine (j)

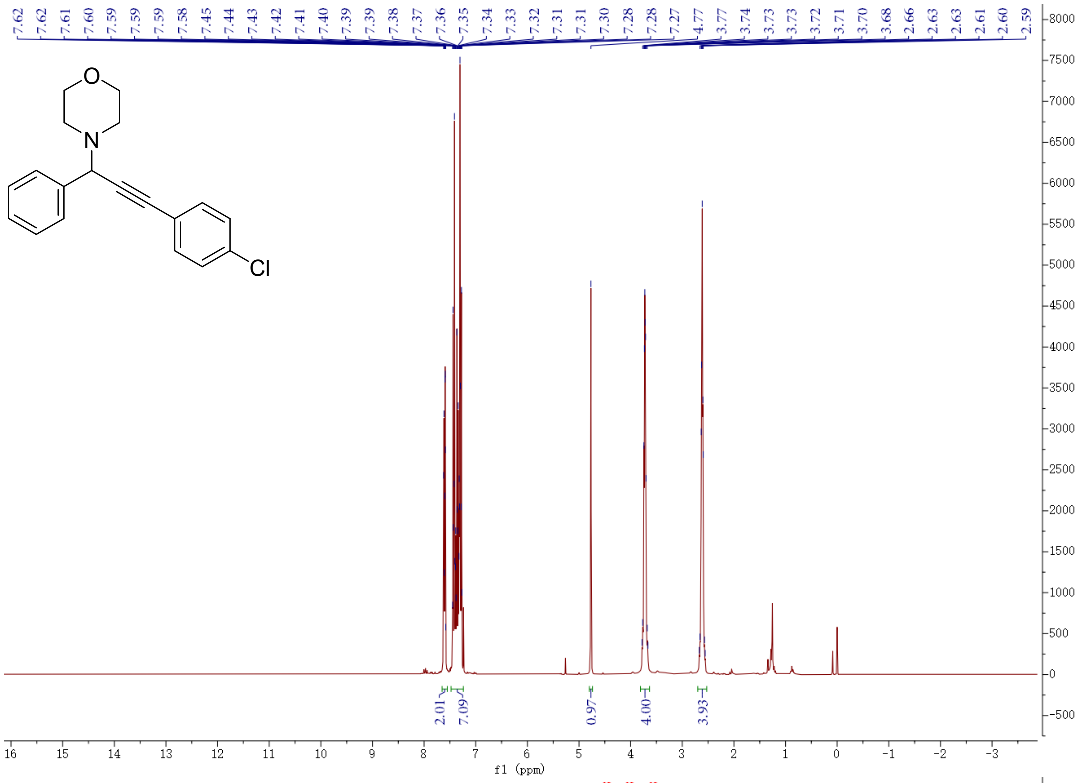

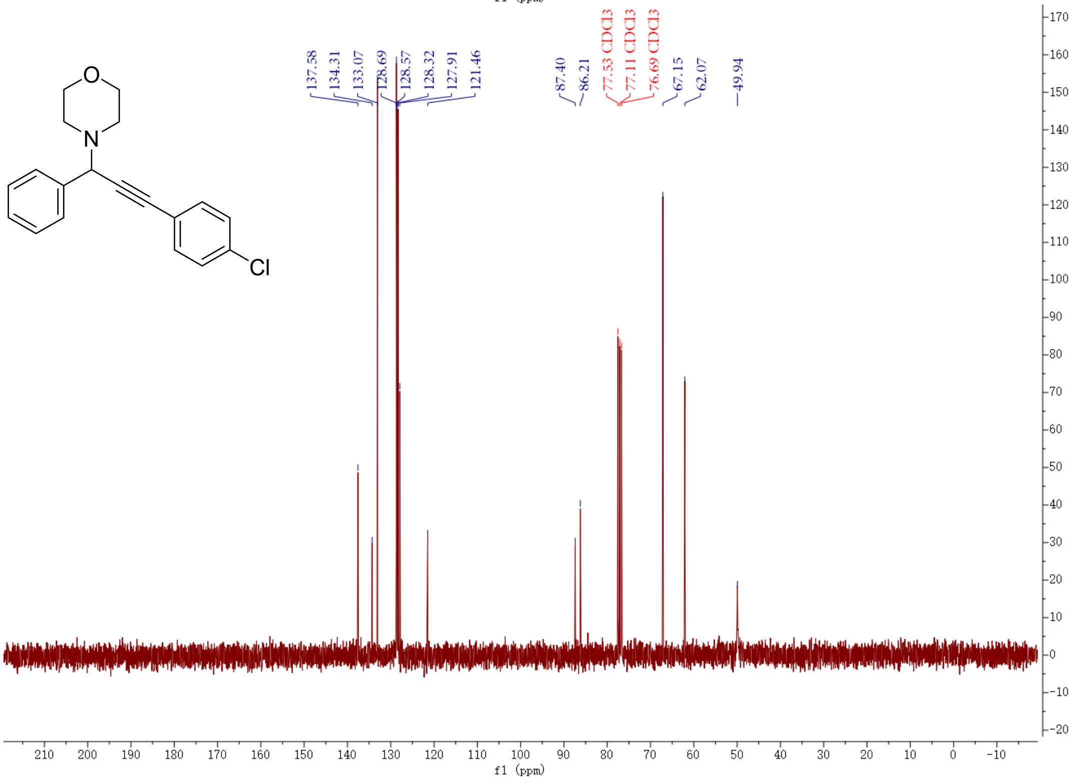

1-(1,3-diphenyl-2-propyn-1-yl)-piperidine (l)

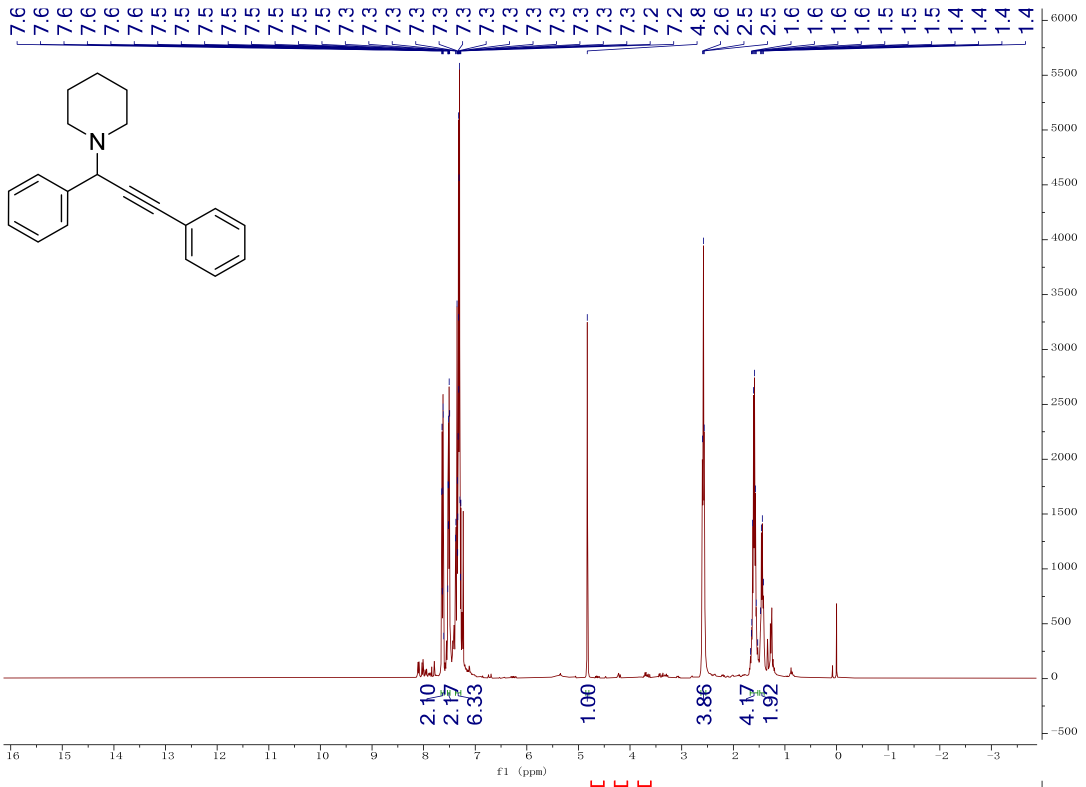

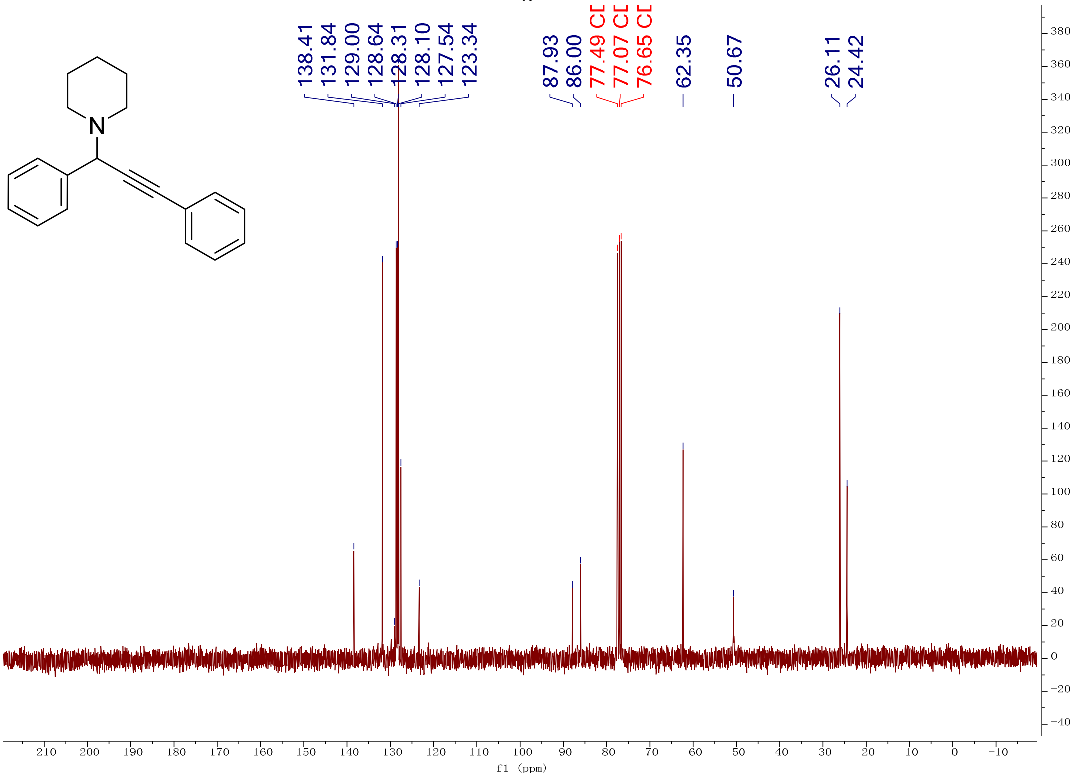

1-[1-(4-Chlorophenyl)-3-phenyl-2-propyn-1-yl]piperidine (m)

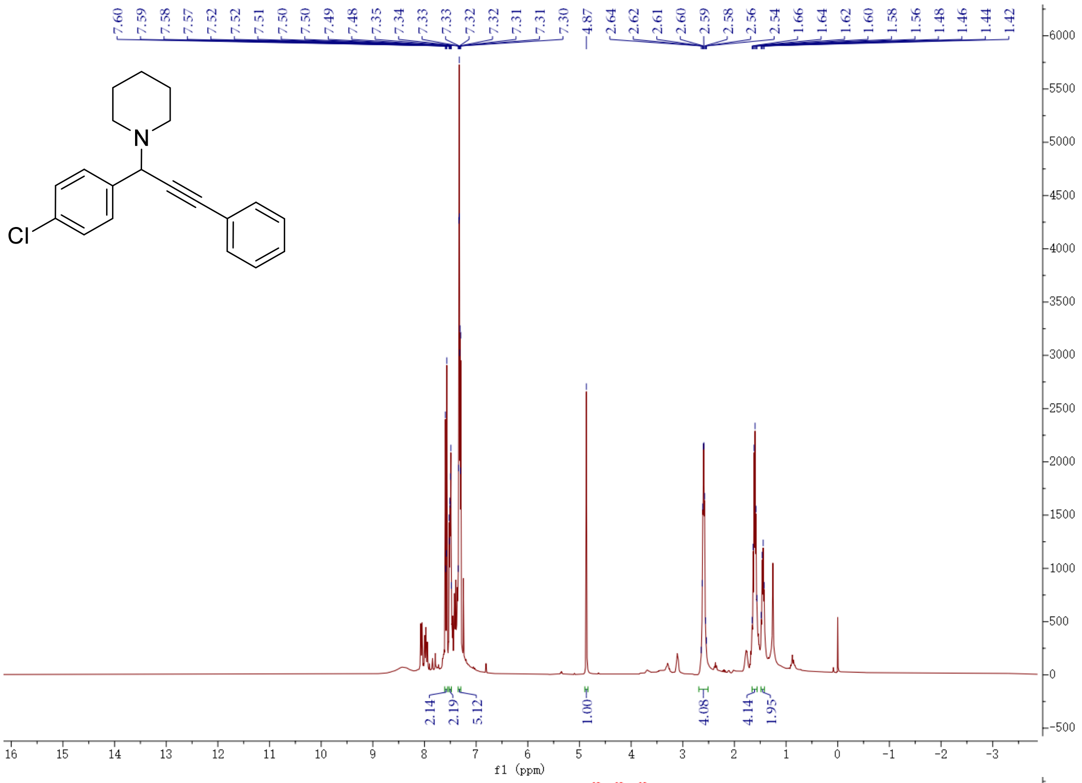

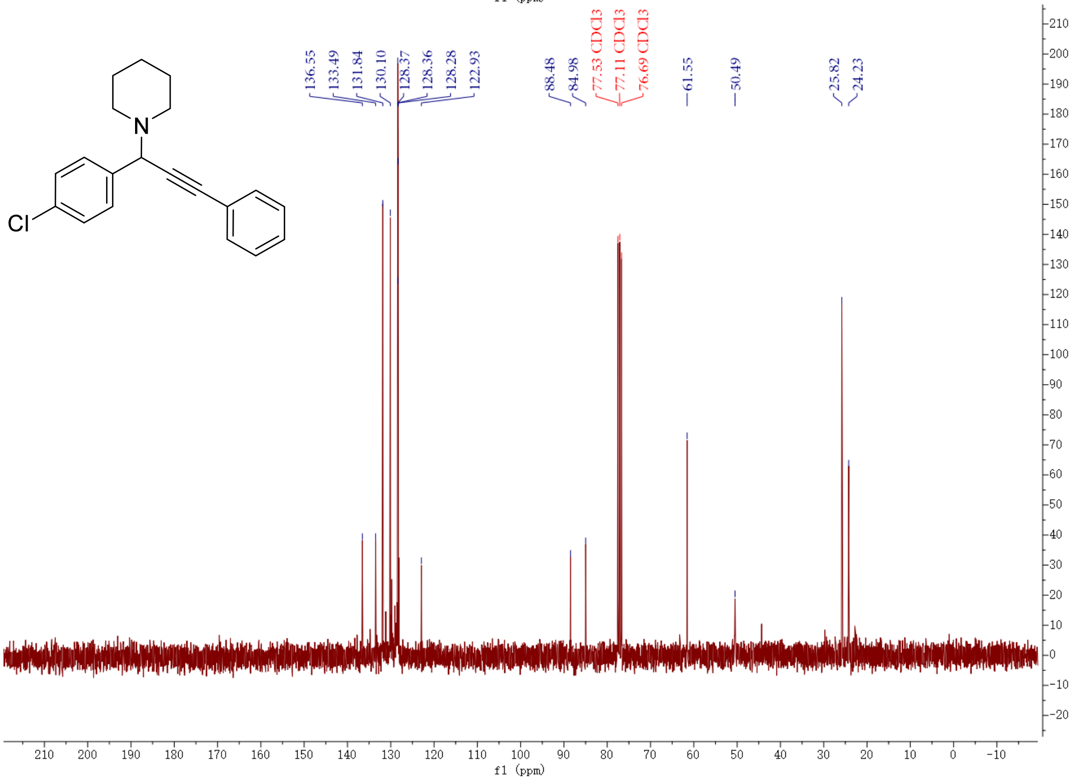

4-Chloro-N, N-diethyl-α-(2-phenylethynyl)benzenemethanamine (n)

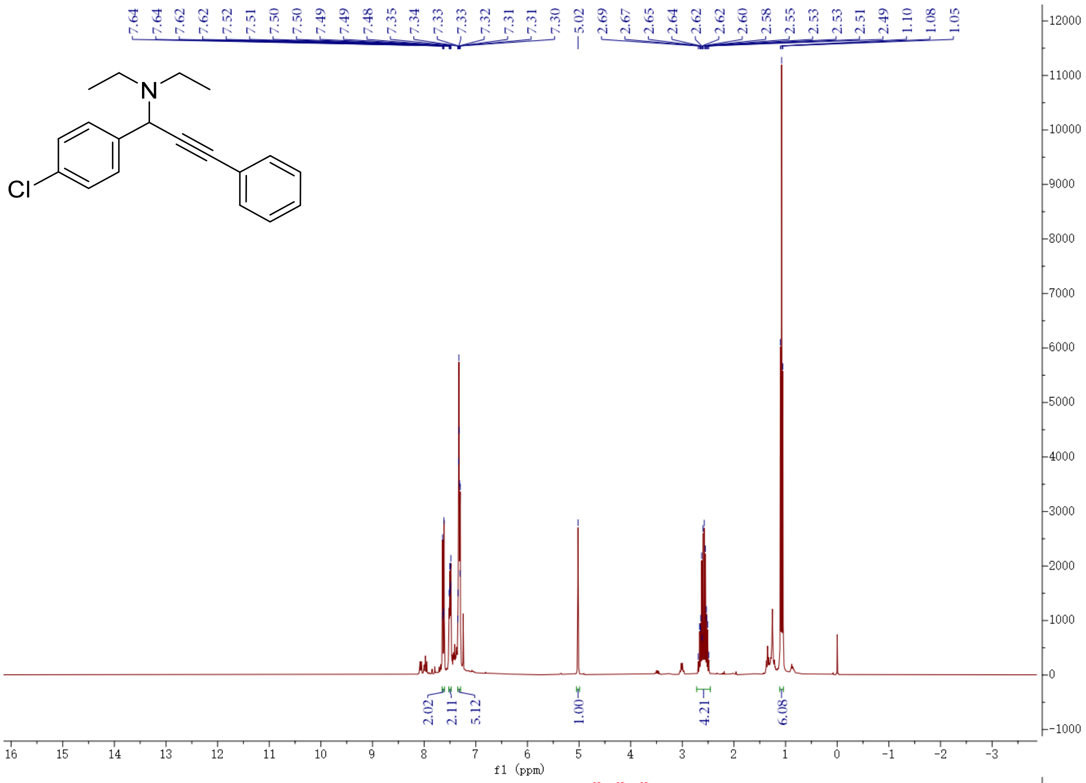

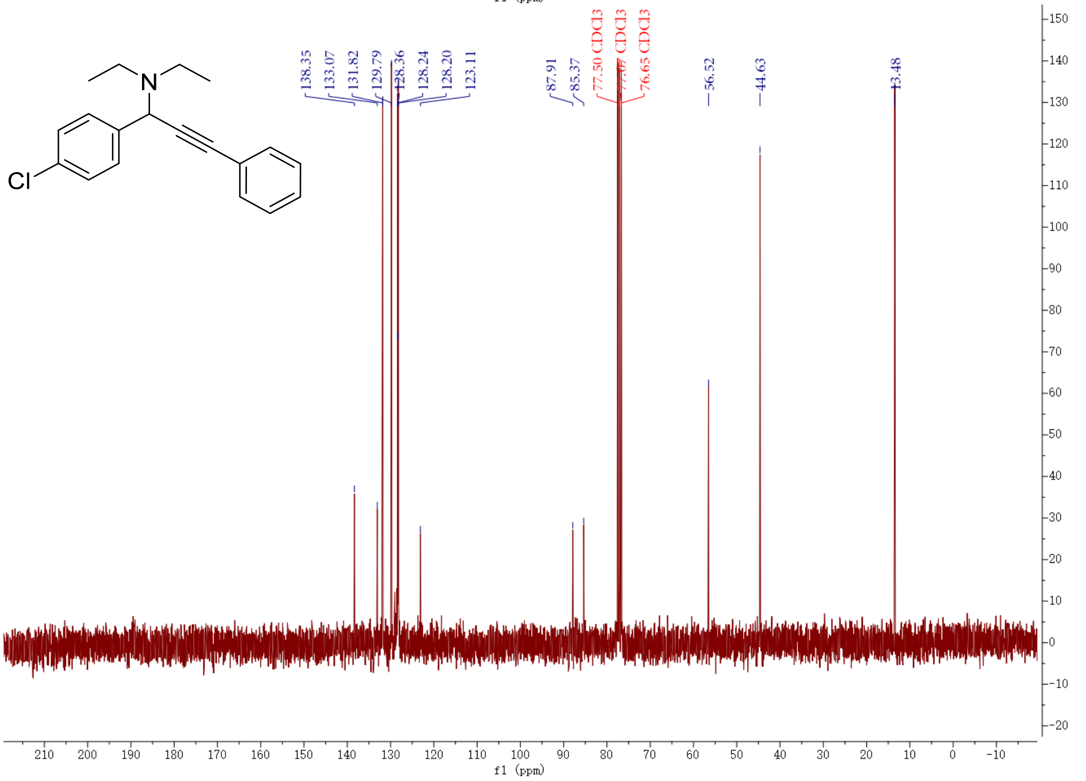

4-(1-Ethyl-3-phenyl-2-propyn-1-yl)morpholine (p)

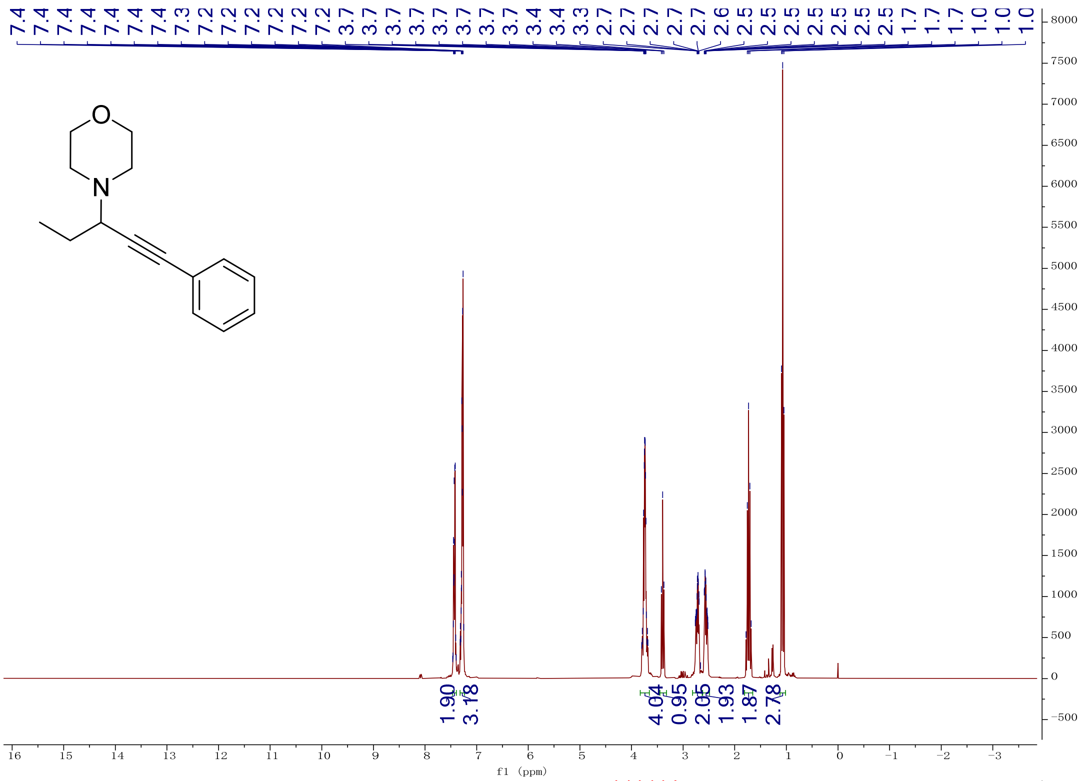

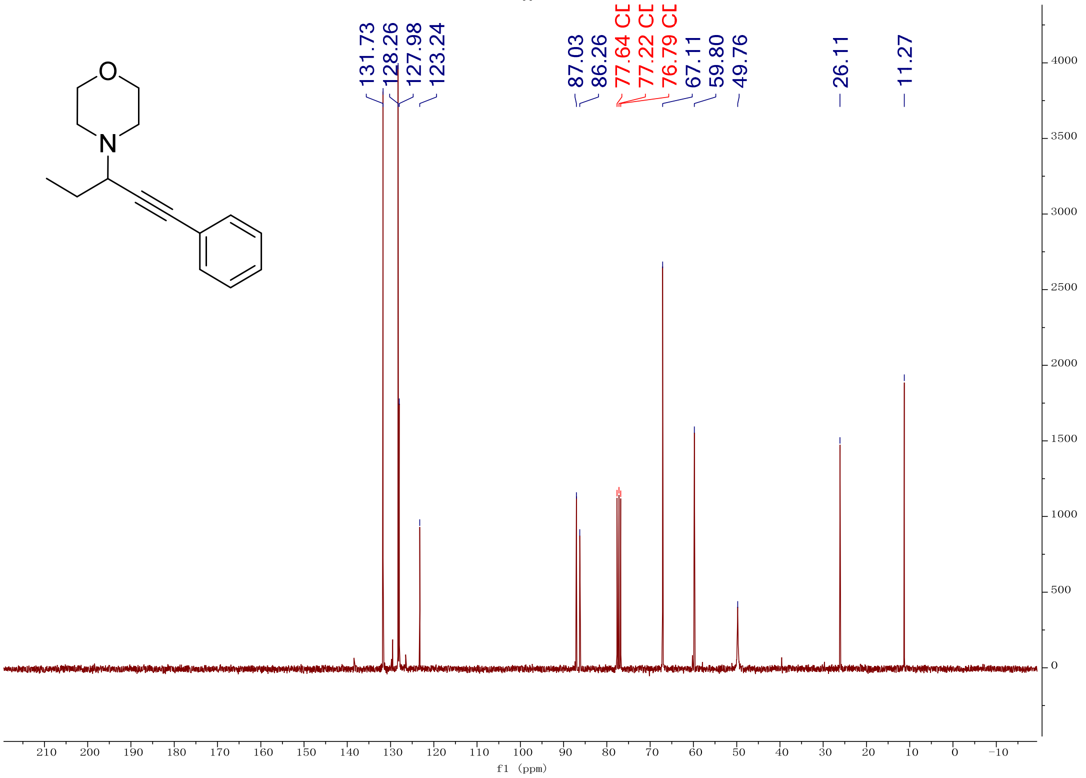

4-[1-(1-Methylethyl)-3-phenyl-2-propyn-1-yl]morpholine (q)

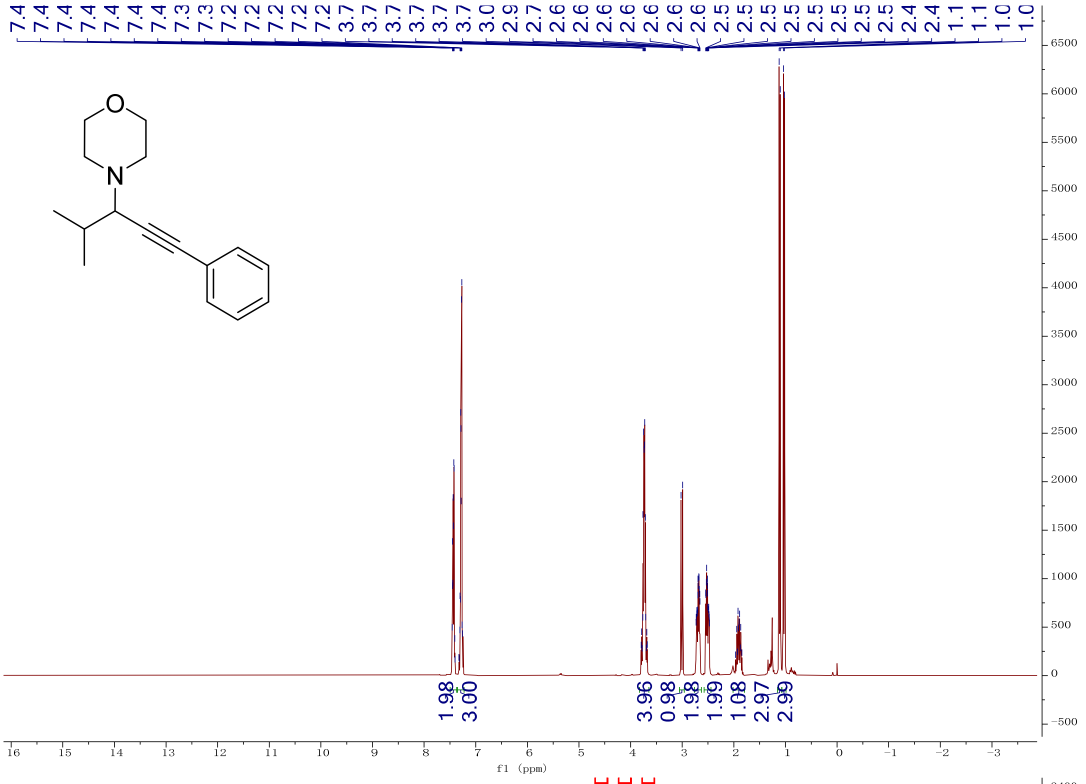

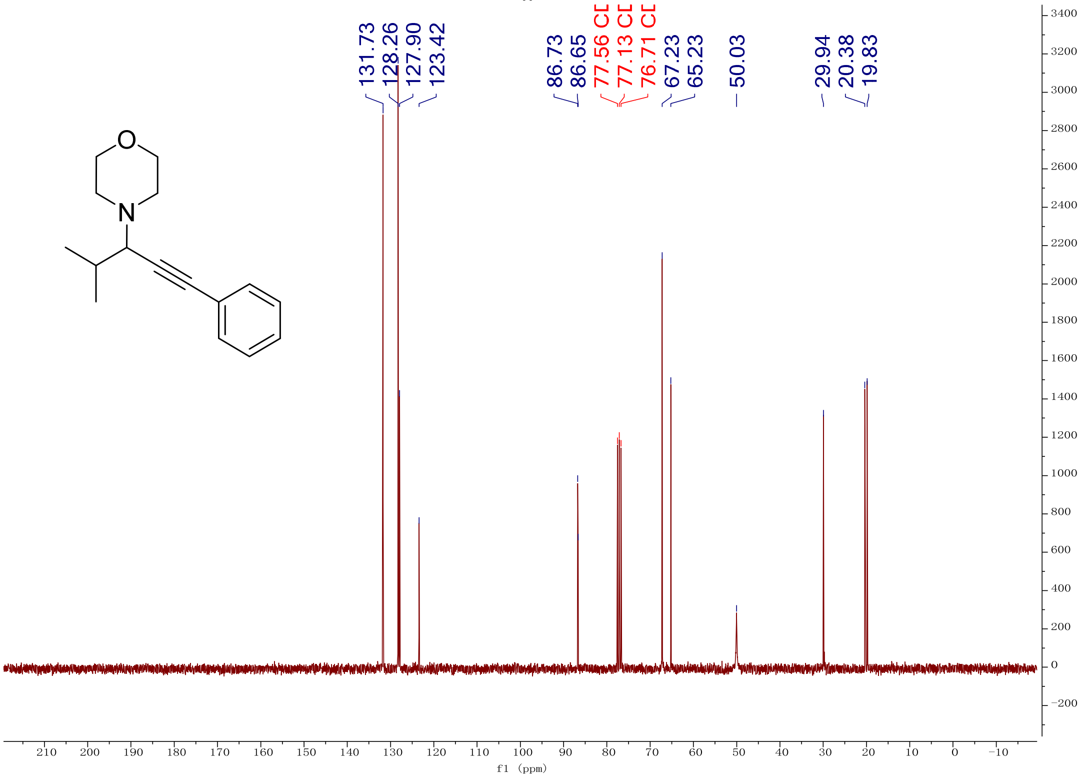

3. Copies of [19] F NMR for synthesized organofluoride

4-[3-[4-(trifluoromethyl)-1-phenyl-2-propyn-1-yl]-2-propyn-1-yl]-morpholine (i)

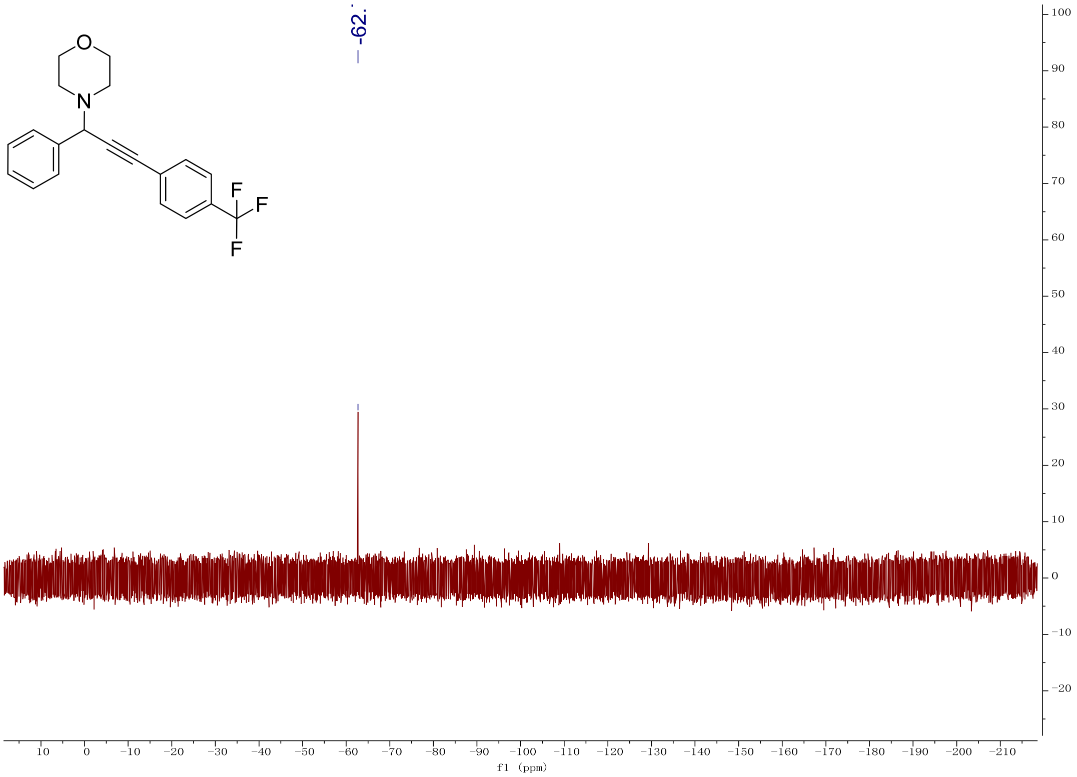

4. Copies of FT-IR and HRMS for new compound

4-[3-[4-(trifluoromethyl)-1-phenyl-2-propyn-1-yl]-2-propyn-1-yl]-morpholine (i)

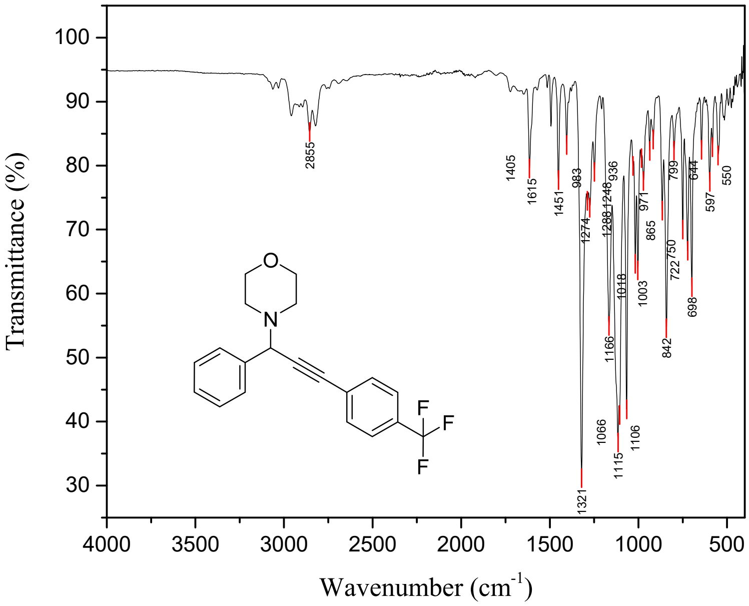

**Sample Name**	XZA--11P	**Position**	P2A4	**Instrument Name**	Instrument 1
**User Name**		**Inj Vol**	10	**InjPosition**	
**Sample Type**	Sample	**IRM Calibration Status**	Success	**Data Filename**	XZA--11P.d
**ACQ Method**	lyq-Positive-Methanol.m	**Comment**		**Acquired Time**	12/31/2021 11:46:55 AM (UTC+08:00)

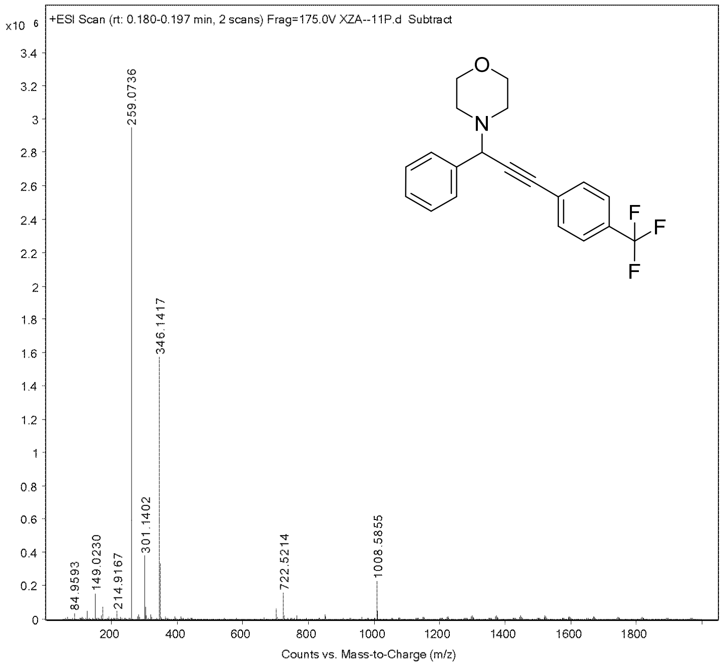

Supplement: Supplementary file 1 [file nanomaterials-12-01641-s001.zip › nanomaterials-1684884-supplementary.pdf]
